# A dual functional theranostic microneedle patch for immunomodulation and real time monitoring in diabetic wound therapy

**DOI:** 10.7150/thno.133451

**Published:** 2026-05-01

**Authors:** Shuai Fan, Junlong Zhong, Yeke Chen, Kang Chen, Zhiming Liu, Xinmin Yang, Wen Tan, Wenlong Tang, Wanhui Zhou, Degui Wu, Jiachao Xiong, Zhenhai Zhou, Fanrong Ai, Kai Cao

**Affiliations:** 1 Orthopedic Hospital, The First Affiliated Hospital, Jiangxi Medical College, Nanchang University, Nanchang, Jiangxi 330209, China.; 2 The Key Laboratory of Spine and Spinal Cord Disease of Jiangxi Province, Nanchang, Jiangxi 330006, China.; 3 Bio 3D Printing Laboratory, School of Advanced Manufacturing, Nanchang University, Nanchang, Jiangxi 330031, China.; 4 Department of Orthopedics, Affiliated Rehabilitation Hospital of Nanchang University, Jiangxi 330003, China.; 5 The Second Clinical Medical School, Nanchang University, Nanchang, Jiangxi 33006, China.

**Keywords:** diabetic wound healing, microneedle patches, metal-organic frameworks, macrophage reprogramming

## Abstract

**Rationale:**

The management of diabetic wound is limited by the absence of delivery systems that can dynamically respond to the complex pathological microenvironment. Herein, we have engineered a dual-functional theranostic microneedle (MN) patch for intelligent diabetic wound therapy.

**Methods:**

The patch (termed MNs@Z/CP) features a spatially designed bilayer architecture: the needle tips are loaded with a catalytic nanozyme (ZTCG) for on-demand therapy, exhibiting cascade superoxide dismutase (SOD)- and catalase (CAT)-mimetic activities to simultaneously alleviate oxidative stress and hypoxia while combating bacterial infection; the backing layer incorporates a cerium metal-organic framework (Ce-MOF)-based visual sensor for real-time monitoring of wound H_2_O_2_ levels.

**Results:**

MNs@Z/CP not only exhibited multimodal antibacterial and anti-inflammatory effects but also reprogrammed the immune microenvironment by activating the Nrf2/HO-1 pathway to shift macrophages from a pro-inflammatory (M1) to a pro-healing (M2) phenotype. In both diabetic and methicillin-resistant *Staphylococcus aureus (MRSA)*-infected diabetic wound models, the patch significantly accelerated wound closure, promoting angiogenesis, collagen deposition, and re-epithelialization.

**Conclusion:**

This work pioneers a theranostic platform that integrates real-time diagnostic, controlled catalytic therapy, and immunomodulatory therapy, providing a viable approach to the autonomous management of chronic wounds.

## Introduction

Diabetes is a global health challenge characterized by persistent hyperglycemia resulting from insulin deficiency or dysfunctional insulin action 1. Diabetic ulcers are among the most common and therapeutically challenging complications of diabetes, affecting approximately 19–34% of diabetic patients 2. These ulcers are linked to high recurrence rates and significant disability, imposing a tremendous strain on both patients and healthcare systems 3. Unlike normal wound healing, diabetic ulcers remain arrested in a persistent inflammation-infection phase, failing to progress through the normal tissue repair program 4.

The hyperglycemic microenvironment is a pivotal driver of poor diabetic wound healing 5. Persistent hyperglycemia induces oxidative stress, leading to the massive accumulation of reactive oxygen species (ROS), including hydrogen peroxide (H_2_O_2_), hydroxyl radicals (^•^OH), and superoxide anions (^•^O_2_^-^), thereby inducing cellular damage and DNA strand breaks 6, 7. Concurrently, the hyperglycemic microenvironment alters the local immune status, promoting the adhesion, colonization, and biofilm formation of pathogenic microorganisms 8. These extracellular polymeric substances-based biofilm structures further enhance bacterial resistance to antibiotics, while the persistent release of metabolic toxins from bacteria exacerbates tissue damage, thereby forming a vicious cycle of "infection-inflammation-tissue damage" 9. In addition, diabetic wounds exhibit dysfunction of the endogenous antioxidant defense system, such as the Nrf2 signaling pathway 10. This weakens the body's ability to clear excess ROS, thereby affecting key repair processes like cell proliferation, macrophage polarization, and angiogenesis 11. Notably, there is a significant synergistic amplification effect between inflammation and oxidative stress: inflammatory responses promote H_2_O_2_ generation, and H_2_O_2_ accumulation further activates inflammatory pathways, establishing a positive feedback loop 12. Consequently, developing comprehensive treatment strategies capable of simultaneously mitigating oxidative stress and dynamically monitoring inflammation is crucial for diabetic wound management.

Metal-Organic Frameworks (MOFs) have been widely used in wound healing applications due to their high specific surface area, tunable porosity, and excellent biocompatibility 13-15. However, conventional MOFs often exhibit limited functionality and insufficient intrinsic antibacterial activity. Therefore, they fail to meet the complex demands of diabetic wound therapy, which requires efficient antibacterial effects, antioxidant stress capacity, and pro-angiogenic activity 16, 17. Passive dressings commonly used in clinical practice do not provide real-time feedback on wound status, making the treatment process a "black box" and impeding precise intervention 18. Although various H_2_O_2_ monitoring technologies based on quantum dots, MOF-based sensors, and upconversion nanoparticles have recently emerged, most of these systems lack the capability to integrate monitoring functions with therapeutic functions 19, 20. Advances in smart wound microneedle patches offer a promising approach to overcome these restrictions 21, 22. By constructing a dual-functional system that combines monitoring and treatment, it becomes possible to intervene in the wound microenvironment and significantly enhance healing efficiency. Among the numerous measurable biochemical indicators, H_2_O_2_ serves as a key molecule that reflects oxidative stress and inflammation levels, providing essential dynamic information for assessing wound status and therapeutic efficacy 23, 24.

**Scheme 1:**
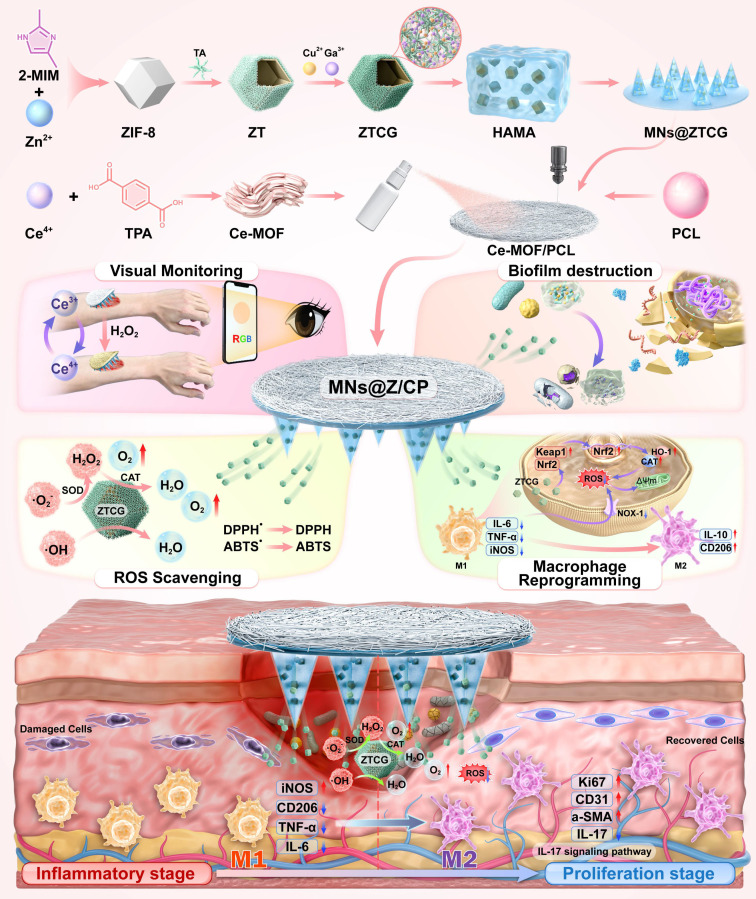
The schematic illustrates the fabrication process of the MNs@Z/CP patch and its mechanism for accelerating diabetic wound healing, which involves H_2_O_2_ visual monitoring, ROS scavenging, biofilm disruption, and macrophage reprogramming.

In this study, we designed a dual-MOF-based bilayer multifunctional microneedle (MN) patch (MNs@Z/CP) (Scheme 1). This system integrated multi-enzyme catalysis, efficient antibacterial activity, macrophage reprogramming, and real-time H_2_O_2_ monitoring to achieve synergistic therapy and management of diabetic wounds. The needle layer was composed of hyaluronic acid methacryloyl (HAMA) hydrogel loaded with ZTCG nanozyme, exhibiting cascade catalase (CAT)- and superoxide dismutase (SOD)-mimetic catalytic activities and potent antibacterial capability. The backing layer consisted of polycaprolactone (PCL) electrospun fibers coated with Ce-MOF (CP), enabling visual monitoring of wound status through its colorimetric response to H_2_O_2_ 25. By triggering the endogenous Nrf2/HO-1 signaling pathway, MNs@Z/CP polarizes macrophages toward the M2 phenotype *in vitro*. This reduced oxidative damage and encouraged cell migration and proliferation. *In vivo* experiments further confirmed that MNs@Z/CP effectively promoted angiogenesis, collagen deposition, and re-epithelialization, while simultaneously suppressing excessive inflammation and inducing M2 macrophage polarization to remodel the healing microenvironment. Transcriptomic analysis revealed that MNs@Z/CP significantly accelerated diabetic wound healing through the synergistic "anti-inflammatory and pro-proliferative" effects. Moreover, the system demonstrated excellent therapeutic outcomes in a methicillin-resistant *Staphylococcus aureus* (*MRSA*)-infected diabetic wound model. This study provided a novel and comprehensive intelligent solution for chronic wounds by integrating regulation of the microenvironment with multi-enzyme catalytic therapy and real-time monitoring capability.

## Materials and Methods

### Materials

Zinc nitrate hexahydrate (Zn(NO_3_)_2_·6H_2_O), 2-methylimidazole (2-MIM), tannic acid (TA), ceric ammonium nitrate ((NH_4_)_2_Ce(NO_3_)_6_), riboflavin, methionine, chloroform, and terephthalic acid (TPA) were purchased from Aladdin. Nitrotetrazolium blue chloride (NBT), copper chloride dihydrate (CuCl_2_·2H_2_O), gallium nitrate, dimethylformamide (DMF) and ferrous sulfate (FeSO4) were purchased from Macklin. Poly(ε-caprolactone) (PCL) was sourced from Sigma-Aldrich. Hyaluronic acid methacryloyl (HAMA) was purchased from EFL. The specialized medium for HUVECs was procured from Sciencell.

### Characterization

The morphological features were characterized by transmission electron microscope (TEM, FEI Talos F200S) and scanning electron microscope (SEM, Apreo 2S Hivac, Thermo Scientific). Crystal structures were investigated by X-ray diffraction (XRD, D8 Advance, Bruker) and X-ray photoelectron spectroscopy (XPS, ESCALAB250Xi, Thermo Scientific). Fourier transform infrared (FTIR) spectra were recorded using an FTIR spectrometer (Nicolet iS20, USA). The hydrodynamic size and zeta potential were determined by zetasizer nano ZS90 (Malvern Instruments). Mechanical testing was conducted on a universal testing machine (CMT6104). Electron spin resonance (ESR/EPR) spectra were acquired using a Bruker EMXplus-6/1 spectrometer. Flow cytometry data were collected using a FacsCalibur cell analyzer.

### Synthesis of ZTCG

Briefly, aqueous solutions of 2-MIM (2.2 g in 10 mL water) and Zn(NO_3_)_2_·6H_2_O (2 mL, 100 mg/mL) were combined and stirred for 15 min. ZIF-8 was recovered via centrifugation 26. After ZIF-8 was dispersed in deionized water (10 mg/mL), 10 mL of TA solution (10 mg/mL) was added, and the mixture was stirred for 10 min. The resulting ZT nanoparticles were collected and redispersed in 10 mL of deionized water. Subsequently, the ZT dispersion was combined with 400 μL of CuCl_2_·2H_2_O (10 mg/mL) and 2 mL of gallium nitrate (10 mg/mL) under stirring. Centrifugation was used to separate the final ZTCG product following a 10-min reaction.

### Synthesis of Ce-MOF and OXCe-MOF

TPA (0.146 g) was dissolved in 2.5 mL of DMF, and 250 μL of triethylamine was added to enhance solubility 27. Separately, (NH_4_)_2_Ce(NO_3_)_6_ (1.000 g) was dissolved in 4 mL of DMF. The TPA solution was then combined with the cerium salt solution under stirring. After 24 h of reaction, Ce-MOF was collected by centrifugation. The Ce-MOF oxidized by hydrogen peroxide is denoted as OXCe-MOF.

### Fabrication of bilayer microneedles (MNs)

First, Ce-MOF-loaded electrospun fibers were prepared. Briefly, PCL (18 wt%) was dissolved in 10 mL of chloroform. The spinning solution was then loaded into a syringe. The PCL electrospun fiber membrane was fabricated at room temperature using the following parameters: an applied voltage of 10 kV, a solution flow rate of 0.8 mL/h, and a collection distance of 13 cm. An ethanol solution of Ce-MOF (2 mg/mL) was sprayed onto the PCL membrane surface three times. After drying for 24 h, the Ce-MOF/PCL (CP) electrospun membrane was obtained.

The ZTCG-HAMA precursor solution was prepared by dispersing ZTCG (200 μg/mL) into a HAMA solution (5% w/v) followed by the addition of a 0.25% w/v lithium phenyl-2,4,6-trimethylbenzoylphosphinate (LAP) solution. Then, 300 μL of the ZTCG-HAMA precursor solution was cast into a polydimethylsiloxane (PDMS) mold. After centrifugation and vacuum degassing, the ZTCG-HAMA (MNs@Z) was dried at 30 °C for 6 h. Subsequently, the CP fibrous mat was attached as the backing layer. The MNs@Z/CP was then polymerized and demolded. Blank HAMA hydrogel microneedles (MNs) with PCL film (P) were denoted as MNs/P, while ZTCG-loaded HAMA hydrogel microneedles (MNs@Z) with PCL film (P) were denoted as MNs@Z/P.

### ROS-responsive drug release

ZTCG samples (200 μg/mL) were incubated in 5 mL of phosphate-buffered saline (PBS) with or without H_2_O_2_ (300 μM). At predefined intervals (3, 6, 12, and 24 h), samples were collected for examination. The release of Cu^2+^ and Ga^3+^ was quantified using inductively coupled plasma optical emission spectrometry (ICP-OES).

### ^•^O_2_^-^ scavenging assay

The inhibition rate of NBT photoreduction was used to assess the ZTCG nanozymes' capacity to scavenge ^•^O_2_^-^. ZTCG nanozymes (0–200 μg/mL) were mixed with 20 μM riboflavin, 12.5 mM methionine, and 75 μM NBT. Following 15 min of UV irradiation, ^•^O_2_^-^ reduced NBT to blue formazan. The absorbance was measured by Ultraviolet-Visible (UV-Vis, Lambda 1050+, PerkinElmer) at 560 nm.

### ^•^OH scavenging assay

The ^•^OH scavenging effectiveness was ascertained using a salicylic acid (SA) trapping method. Briefly, ^•^OH radicals were generated via the Fenton reaction between H_2_O_2_ (2 × 10^-4^ M) and FeSO_4_ (2 × 10^-4^ M). ZTCG nanozymes (0-200 μg/mL) were added to scavenge the ^•^OH. Subsequently, SA (1×10^-3^ M) was added to react with the residual ^•^OH, forming purple-colored 2,3-dihydroxybenzoic acid. The absorbance was measured at 510 nm.

### ABTS^•^ and DPPH^•^ scavenging assays

In short, the ABTS^•^ cation was generated by mixing potassium persulfate (2.6 mM, 1 mL) with ABTS (7.4 mM, 1 mL) and leaving the mixture in the dark for 16 h. PBS was used to dilute the resultant ABTS^•^ solution 30-fold. ZTCG nanozymes (0-200 μg/mL) were dispersed in the ABTS^•^ solution, and the combination was then kept in the dark for 20 min. Absorbance was measured at 734 nm. The ABTS^•^ scavenging rate was calculated using the formula: Scavenging (%) = (A_0_ - A) / A_0_ × 100%, where A_0_ and A are the absorbance values without and with ZTCG.

To test the DPPH^•^ scavenging activity, ZTCG nanozymes (0-200 μg/mL) were reacted with DPPH^•^ solution in dark for 5 min. At 517 nm, the absorbance was measured. The DPPH^•^ scavenging rate was calculated using the formula: Scavenging (%) = (A_0_ - A) / A_0_ × 100%, where A_0_ and A are the absorbance values without and with ZTCG.

### CAT-like activity of ZTCG nanozymes

The ZTCG nanozyme (0–200 μg/mL) was mixed with H_2_O_2_ in PBS (0.1 M) to evaluate its CAT-like activity. The oxygen (O_2_) generated was quantified using a dissolved oxygen meter. Additionally, the H_2_O_2_ scavenging activity of ZTCG was measured by commercial hydrogen peroxide assay kit (Solarbio).

### Electrochemical measurements of ZTCG nanozymes

Cyclic voltammetry (CV) was measured in PBS with or without H_2_O_2_ (300 μg/mL) at a scan rate of 50 mV/s by an electrochemical workstation (CHI 760E, China). A standard three-electrode system was employed, with a glassy carbon electrode modified by ZTCG serving as the working electrode, a platinum wire serving as the counter electrode, and an Ag/AgCl electrode as the reference electrode.

### Antibacterial activity of ZTCG nanozymes

*MRSA* (ATCC33591) and *Escherichia coli* (*E. coli*, ATCC25922) were selected as model strains. ZTCG nanozymes (0-200 μg/mL) were co-cultured with bacterial suspension (2 mL, 10^8^ CFU/mL) for 6 h. After co-culture, the suspensions were diluted 10^-4^-fold and plated on agar plates for measurement. For bacterial morphology observation, the co-cultured bacterial suspensions were fixed, dehydrated, freeze-dried, and observed by SEM and TEM.

### Biofilm eradication assay of ZTCG nanozymes

To create *E. coli* and *MRSA* biofilms, cell culture slides and bacterial (10^8^ CFU/mL, 2 mL) were added in 12-well plates for three days. Following 24 h treatment with various concentrations of ZTCG solution, the pre-formed biofilms were rinsed twice with PBS. The biofilms were stained with 0.1% crystal violet solution and examined by optical microscope. The absorbance (OD) at 595 nm was determined after the stained biofilms were solubilized in ethanol. Elimination rate (%) = (OD_0_ - OD_Z_) / OD_0_ × 100%, where OD_0_ and OD_Z_ represent the OD values without and with ZTCG treatment, respectively. Additionally, the biofilms were stained with Bacterial Live/Dead Staining Kit (Beyotime) and imaged with a laser scanning confocal microscope (CLSM, OLYMPUS FV1000).

### Bacterial membrane permeability of ZTCG nanozymes

The membrane permeability of *MRSA* biofilms was measured using the o-nitrophenyl-β-D-galactopyranoside (ONPG) hydrolysis experiment. In summary, *MRSA* biofilms were exposed to various concentrations of ZTCG nanozymes for 24 h. Following centrifugation, the supernatant was examined by an ONPG test kit (Solarbio, Beijing, China). The absorbance of the supernatant was measured at 420 nm.

### Protein leakage

After co-incubating different concentrations of the ZTCG nanozyme with the MRSA suspension for 48 h, the mixture was centrifuged at 5000 rpm (4 °C, 10 min) to obtain the supernatant. Protein leakage was quantified using a BCA protein assay kit (Beyotime).

### Bacterial membrane potential detection

The DiSC_3_(5) fluorescent probe was used to measure bacterial membrane potential. After co-culturing of *MRSA* suspensions (10^6^ CFU/mL) with various concentrations of ZTCG nanozymes, the fluorescence signal was visualized under a CLSM.

### Cytocompatibility assay

In 24-well plates, L929 fibroblasts and HUVECs (2 × 10^4^) were seeded and treated with various MNs. Cell viability was assessed using CCK-8 reagent (Solarbio, China), and cell morphology was observed by Calcein-AM/PI staining (dark, 20 min) on days 1 and 3.

### Intracellular ROS scavenging

The intracellular ROS scavenging capacity of MNs@Z/CP was evaluated using a hydrogen peroxide-induced oxidative stress model. After seeding in 24-well plates, L929 cells were cultivated for 24 h, then exposed to MNs/P, MNs@Z/P, and MNs@Z/CP in 300 μM H_2_O_2_ for 24 h. After stained with DCFH-DA (5 μM), the cells cleaned with PBS and observed under a fluorescence microscopy. Cell viability under oxidative stress was separately performed using Calcein-AM/PI staining and CCK-8 assay.

### Cell proliferation assays

In the EdU assay, L929 cells were treated with MNs/P, MNs@Z/P, and MNs@Z/CP along with H_2_O_2_ (300 μM) for 24 h, followed by the addition of EdU (10 μM) and further incubation for 12 h. Following the kit's instructions for fixation, permeabilization, blocking, and staining, fluorescence microscopy was used to obtain fluorescence pictures.

### Wound scratch assay

After seeding L929 cells (2 × 10^5^) in 24-well plates, a linear scratch wound was created using a sterile 200 μL pipette tip. The experimental groups were treated with MNs/P, MNs@Z/P, and MNs@Z/CP along with H_2_O_2_ (300 μM). Microscopy was used to take pictures of the scratch wounds, and quantitative analysis was performed with ImageJ.

### Tube formation assay

Matrigel (50 μL) was added to 96-well plates and polymerized at 37 °C (30 min). HUVECs (1 × 10^4^) were seeded onto the Matrigel and exposed to MNs/P, MNs@Z/P, and MNs@Z/CP along with H_2_O_2_ (300 μM) for 6 h. Images were acquired using a microscope and quantitatively analyzed with ImageJ.

### Intracellular ROS scavenging by MNs@Z/CP in cells

In 24-well plates, RAW 264.7 macrophages (2 × 10^4^) were seeded and stimulated with LPS (100 ng/mL, 12 h). After that, the cells were co-cultured with MNs/P, MNs@Z/P, and MNs@Z/CP for 24 h. The cells staining was performed using 5 μM DCFH-DA, followed by PBS washes and fluorescence microscopic imaging.

### Mitochondrial membrane potential (ΔΨ_m_) assay

After treatment with LPS and the materials, cells were stained for 30 min (37 °C, dark) using 5 μM JC-1 solution. After washing with PBS, the cells were observed under CLSM.

### Evaluation of macrophage polarization phenotypes

Following a 12-h stimulation with LPS (100 ng/mL), the macrophages were treated with MNs/P, MNs@Z/P, and MNs@Z/CP for 24 h. Following their fixation in 4% paraformaldehydeand processed according to a standard immunofluorescence protocol. This included blocking, incubation with iNOS and CD206 primary antibodies (4 °C, overnight), and subsequent treatment with secondary antibodies for 30 min. Nuclei were delineated using DAPI staining. Imaging was performed using a CLSM, quantitative analysis of the fluorescence signal was carried out with ImageJ.

For real-time quantitative polymerase chain reaction (RT-qPCR), TRIzol reagent was used to extract total RNA from treated macrophages. After reverse transcription into complementary DNA, the mRNA expression levels of* iNOS*, *IL-6*, *TNF-α*,* CD206*, and *IL-10* were determined. The primer sequences are listed in Table S1. Western blot (WB) was employed to detect iNOS and CD206 protein expression and quantified using ImageJ. The primary antibodies are listed in Table S2.

### *In vitro* anti-inflammatory mechanism of MNs

Immunofluorescence staining was used to assess Nrf2 and HO-1 expression. Meanwhile, the expression levels of Nrf2, KEAP1, HO-1, CAT, and NOX1 were assessed by RT-qPCR and WB analysis.

### Animal models and treatments

All animal procedures were conducted in strict compliance with the national guidelines for animal welfare and ethical review and were approved by the Animal Ethics Committee of Nanchang University (Nanchang, China, Approval No. NCULAE-202209280028). Sprague-Dawley rats (male, 8-week-old) were purchased from Changsha Tianqin Biotechnology Co., Ltd. Streptozotocin (STZ, 45 mg/kg) injections were used to cause diabetes following a week of acclimation. The rats were monitored daily. The tail vein was used to assess blood glucose levels at random time points. Sustained elevated blood glucose (>16.7 mmol/L) was classified as diabetic and used for subsequent experiments.

Diabetic rats were randomly assigned to four groups (n = 15): blank group, MNs/P group, MNs@Z/P group, and MNs@Z/CP group. A full-thickness skin wound (10 mm diameter) was made on the dorsal skin. The blank group received no treatment, while the other three groups were treated with MNs/P, MNs@Z/P, or MNs@Z/CP patches, respectively. The wound healing process (0, 3, 7 and 12 days) was periodically photographed and quantitatively analyzed by ImageJ software. At the predetermined endpoints, tissue samples were harvested for histological evaluation and transcriptome sequencing.

To produce the *MRSA*-infected diabetic wound model, diabetic rats received full-thickness wounds (10 mm in diameter), followed by injection of 50 μL of *MRSA* suspension (10^8^ CFU/mL) into the wound site. The MNs application regimen and experimental groups were consistent with the dorsal wound experiment. The wound healing process (0, 4, 9 and 15 days) was periodically photographed and quantitatively analyzed by ImageJ software. Microbial assessment involved serial dilution and plating of wound exudates collected on postoperative days 4 and 9 on LB agar.

### Histological staining

After fixation, wound tissue samples were embedded in paraffin and cut into approximately 5-μm-thick slices for subsequent H&E, Masson's trichrome, Giemsa, and immunohistochemical and fluorescence staining. Quantitative analysis was performed with ImageJ software.

### *In vivo* biosafety assessment

On day 15, major organs were harvested from rats and stained with H&E to detect any potential abnormalities induced by the MNs@Z/CP treatment.

### Transcriptome sequencing (RNA-Seq)

Wound tissues from the blank group and MNs@Z/CP-treated group (n = 3) of diabetic rats were harvested on postoperative day 12 for RNA sequencing (diabetic wound model). After assessing RNA purity and integrity, the qualified RNA was processed into cDNA libraries and subjected to paired-end sequencing. An Illumina NovaSeq X Plus platform was used for the sequencing process. Differential gene expression analysis, Gene Ontology (GO) enrichment analysis, and Kyoto Encyclopedia of Genes and Genomes (KEGG) pathway analysis were conducted.

### Statistical analysis

At least three independent repetitions of each experiment were conducted, and the date were displayed as mean ± SD. Statistical analyses for all comparative studies in this work was evaluated using GraphPad Prism software (9.2.0). One-way analysis of variance (ANOVA) and *t*-test were used for statistical analyses. Statistical significance is denoted as *p < 0.05, **p < 0.01, and ***p < 0.001.

## Results and Discussion

### Preparation and characterization of ZTCG nanozymes

**Figure 1:**
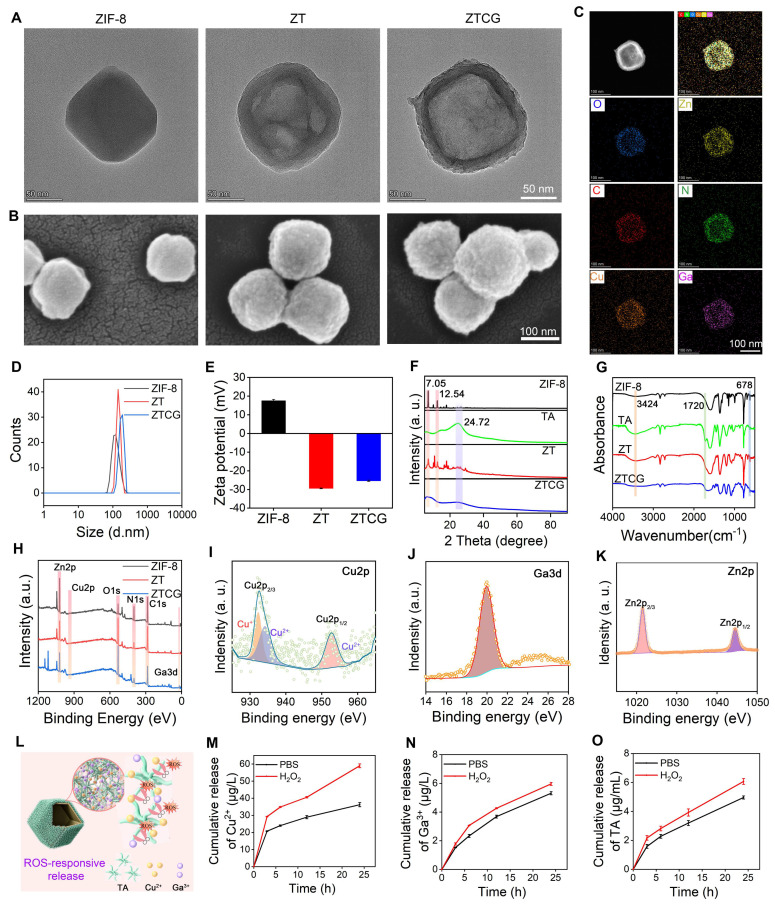
** Preparation and characterization of ZTCG nanozymes.** (A, B) TEM and SEM images of ZIF-8, ZT, and ZTCG. (C) Elemental mapping of ZTCG nanozymes. (D) DLS measurements. (E) Zeta potential analysis (n = 3). (F) XRD patterns. (G) FTIR spectra. (H) XPS survey spectrum of ZTCG nanozymes. (I-K) XPS analysis of Cu 2p, Ga 3d, and Zn 2p in ZTCG nanozymes. (L) Schematic diagram of the ROS-responsive release of ZTCG nanozymes. (M-O) Release profiles of TA, Ga^3+^, and Cu^2+^ from ZTCG with or without H_2_O_2_ treatment. Values are expressed as the mean ± SD.

ZIF-8 nanoparticles were initially synthesized using a one-pot approach 28. As shown in Figure 1A, the as-prepared ZIF-8 nanoparticles exhibited a typical polyhedral morphology. Tannic acid (TA) was then introduced onto their surface to form ZT composites. The TA layer not only provided strong chelating capacity but also etched the ZIF-8 core, transforming it into a hollow spherical structure (Figure 1A) 26. Subsequently, copper (Cu) and gallium (Ga) ions were added to form a stable TA-CuGa metal-phenolic network (MPN). This process ultimately produced the ZTCG nanozymes with a hollow structure (Figure 1A). SEM imaging revealed that the surface of ZTCG was relatively rough, whereas the original ZIF-8 surface was smooth (Figure 1B). The energy dispersive X-ray spectroscopy (EDS) results verified the presence of Zn, Cu, Ga, C, and O in the ZTCG nanozymes (Figure 1C, Figure S1 and S2), confirming successful MPN coating. Dynamic light scattering (DLS) measurements showed that all samples had a hydrodynamic diameter of approximately 150 nm. However, a slight increase in the size of the ZTCG nanozymes was observed after MPN functionalization (Figure 1D). Zeta potential measurements provided further evidence for successful synthesis of ZTCG (Figure 1E). The original ZIF-8 nanoparticles possessed a surface charge of +17.6 ± 0.66 mV. After TA etching, the zeta potential of ZT reversed significantly to -29.4 ± 0.2 mV. Following the addition of Cu^2+^ and Ga^3+^, the zeta potential of ZTCG nanozymes shifted slightly to -25.33 ± 0.35 mV. These changes verified the successful coordination between TA and metal ions.

The XRD pattern in Figure 1F demonstrated that the crystal structure of ZIF-8 was not significantly altered by TA etching alone. The ZT composite retained the characteristic peaks of ZIF-8 at 7.05° and 12.54°, and an additional diffraction peak corresponding to TA appeared at 24.72°. However, the persistent etching effect of TA caused the ZTCG nanozymes to change into an amorphous structure after the MPN shell formed, resulting in the complete disappearance of the characteristic ZIF-8 diffraction peaks. The chemical structure was further characterized by FTIR spectroscopy (Figure 1G). In contrast to ZIF-8, ZT showed a stretching vibration peak at 1720 cm^-1^, which matched the C=O bond in ester groups. The broad peak of ZT in the 3200-3500 cm^-1^ region was much more pronounced, owing to O-H vibrations from the numerous hydroxyl groups in TA 29. Concurrently, encapsulation of the ZIF-8 core by the MPN shell significantly reduced the intensity of the Zn-N vibration peak at 678 cm^-1^ 30. The full-scale XPS survey spectrum (Figure 1H) clearly showed characteristic peaks for C 1s, N 1s, O 1s, Zn 2p, Cu 2p, and Ga 3d, confirming successful modification by the MPN network. The high-resolution Cu 2p spectrum (Figure 1I) showed a mixed valence state of Cu^+^/Cu^2+^, resulting from the reducing capability of TA toward Cu^2+^ during coordination 31. Ga was primarily present as Ga^3+^, and the Zn 2p spectrum corresponded to Zn^2+^ (Figure 1J, K).

Similar to other metal-polyphenol network nanozymes, the mixed valence states of Cu^+^/Cu^2+^ in ZTCG mimic the electron redox behavior of natural enzymes, thereby endowing them with nanozyme characteristics 32, 33. Meanwhile, its MPN structure exhibits ROS-responsive degradation capability, enabling on-demand therapy (Figure 1L). This process produced two biological effects. On the one hand, it can quickly scavenge excess ROS, effectively reducing oxidative stress in the wound microenvironment. On the other hand, it facilitates the release of TA, Cu²⁺, and Ga^3+^, which collectively exert antibacterial, anti-inflammatory, and tissue-repair-promoting effects. To verify the ROS-responsive degradation behavior of this coating, we examined the cumulative release profiles of Cu^2+^, Ga^3+^, and TA under high oxidative stress conditions (300 μM H_2_O_2_) and simulated physiological conditions (PBS). As shown in Figure 1M and 1N, ion release was slow with low cumulative amounts in PBS, whereas the release of both Cu^2+^ and Ga^3+^ was significantly increased in the H_2_O_2_ environment. A similar release behavior was observed for TA (Figure 1O). These results demonstrate that ZTCG nanozymes possess excellent ROS-responsive characteristics, enabling the release of therapeutic factors on demand based on the ROS levels in diabetic chronic wounds.

### Multi-enzyme activities and free radical scavenging capacity of ZTCG nanozymes

**Figure 2:**
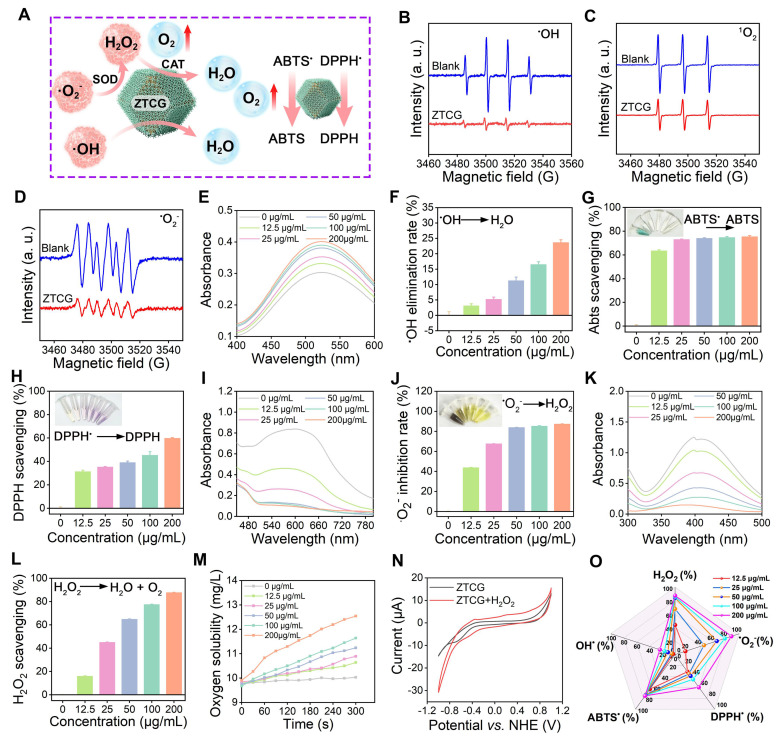
** Multi-enzyme activities and antioxidant capacity of ZTCG nanozymes.** (A) Proposed scavenging mechanisms of ZTCG nanozymes against various free radicals. (B-D) ESR spectroscopy of ZTCG for ^•^OH, ^1^O_2_, and ^•^O_2_^-^. (E, F) ^•^OH scavenging profile (E) and corresponding scavenging efficiency (F) of ZTCG nanozymes. (G, H) Scavenging efficiency of ZTCG nanozymes against ABTS^•^ (G) and DPPH^•^ (H). (I, J) ^•^O_2_^-^ scavenging profile (I) and scavenging efficiency (J) of ZTCG nanozymes. (K, L) H_2_O_2_ scavenging profile (K) and scavenging efficiency (L) of ZTCG nanozymes. (M) O_2_ generation curve of ZTCG nanozymes. (N) CV curves of ZTCG with and without H_2_O_2_. (O) Radar chart illustrating the multi-enzyme activities and radical scavenging capacity of ZTCG nanozymes at various concentrations. Values are expressed as the mean ± SD. (n = 3).

The elevated ROS levels in the diabetic wound microenvironment disrupt redox homeostasis, leading to significant cellular damage and impaired wound healing 34. A key therapeutic strategy currently involves the use of exogenous nanozymes to scavenge excess ROS and restore redox balance. As illustrated in **Figure 2A**, ZTCG nanozymes not only effectively eliminate ^•^O_2_^-^ and H_2_O_2_ through their CAT-like and SOD-like activities, but also directly scavenge various free radicals, including ^•^OH, ABTS^•^, and DPPH^•^.

ESR results clearly showed that the ZTCG nanozymes effectively eliminated ^•^OH, ^1^O_2_, and ^•^O_2_^-^ (Figure 2B-D). We further employed a salicylic acid capture assay to quantitatively measure the removal of ^•^OH. In this assay, ^•^OH radicals oxidize salicylic acid to produce 2,3-dihydroxybenzoic acid, which has a distinct light absorption peak at 510 nm 35. After adding the ZTCG nanozymes, the absorbance at 510 nm decreased sharply in a concentration-dependent manner (Figure 2E). At 200 μg/mL, ZTCG nanozymes achieved a ^•^OH scavenging rate of 23.70 ± 0.85% (Figure 2F).

The broad-spectrum radical scavenging ability of ZTCG nanozymes was evaluated using DPPH^•^ and ABTS^•^ assay kits. In the ABTS^•^ assay, after treatment with ZTCG nanozymes, the solution changed from bluish-green to colorless. At 200 μg/mL, the ABTS^•^ scavenging rate of ZTCG reached 75.47 ± 0.86% (Figure 2G). Similarly, after 15 min of co-incubation, ZTCG nanozymes scavenged 59.84 ± 0.45% of DPPH^•^ at the same concentration, accompanied by a color change from purple to yellow (Figure 2H). These results confirm the excellent free radical scavenging capacity of ZTCG nanozymes.

SOD is one of the key antioxidant enzymes within cells. To investigate the SOD-like activity of ZTCG nanozymes, the NBT photoreduction method was used. Under UV radiation, ^•^O_2_^-^ generated from riboflavin and methionine converts colorless NBT to blue formazan, which exhibits maximal absorbance at 560 nm 35. The addition of ZTCG nanozymes significantly decreased the absorbance of the solution (Figure 2I). The inhibition rate against ^•^O_2_^-^ exceeded 67.66 ± 0.07% at a ZTCG nanozyme concentration of 25 μg/mL, demonstrating excellent SOD-like activity (Figure 2J).

The primary source of oxidative damage in biological systems is H_2_O_2_, which is broken down by the vital enzyme catalase 36. We evaluated the H_2_O_2_ scavenging ability of ZTCG nanozymes using a hydrogen peroxide assay kit. A dose-responsive reduction in absorbance at 410 nm was observed with increasing ZTCG concentrations (Figure 2K, L). Most impressively, at 200 μg/mL, ZTCG nanozymes achieved a remarkable H_2_O_2_ scavenging rate of 87.75 ± 0.25%, confirming their broad ROS scavenging activity. Oxygen is a common product following the SOD/CAT-mimicking activities of nanozymes. To monitor the oxygen generation capacity of ZTCG nanozymes, we performed real-time detection using a dissolved oxygen meter. After incubating different concentrations of ZTCG nanozymes with 1 mM H_2_O_2_ at room temperature, oxygen production increased continuously over time and with increasing nanozyme concentration. At 200 μg/mL, the dissolved oxygen concentration reached 12.54 mg/L within 300 s, whereas almost no oxygen release was detected At 0 μg/mL (Figure 2M and Figure S3). Subsequently, the Michaelis-Menten saturation curve for steady-state kinetic measurements was fitted using varying concentrations of H_2_O_2_ as the substrate. According to the Lineweaver-Burk plot, the maximum reaction velocity (V_max_) and Michaelis-Menten constant (K_m_) were determined to be 1.26 mg L^-1^ min^-1^ and 70.52 mM, respectively, as shown in Figure S4. Based on these kinetic parameters, the CAT-like catalytic activity of the ZTCG nanozymes is higher than those of previously reported nanozymes 37, 38.

To further investigate its CAT-like activity, the electrocatalytic behavior of ZTCG towards H_2_O_2_ was measured using cyclic voltammetry. Figure 2N shows the cyclic voltammetry curves of the ZTCG-modified electrode in PBS with and without 3 mM H_2_O_2_. Upon the addition of H_2_O_2_, the reduction current increased significantly starting from approximately -1 V. Concurrently, the anodic current around 0.65 V also increased. This increase in electron transfer efficiency upon H_2_O_2_ addition demonstrates the compound's potent catalase-like catalytic activity.

As shown in Figure 2O, the multi-enzyme catalytic actions of the ZTCG nanozymes show a clear concentration dependence. This multi-enzymatic catalysis not only exhibits outstanding scavenging activity against various ROS but also reduces local hypoxia in the wound through *in situ* oxygen delivery, offering twofold assurance in reducing cellular oxidative damage and fostering wound healing.

### Antibacterial and anti-biofilm activity of ZTCG nanozymes

**Figure 3:**
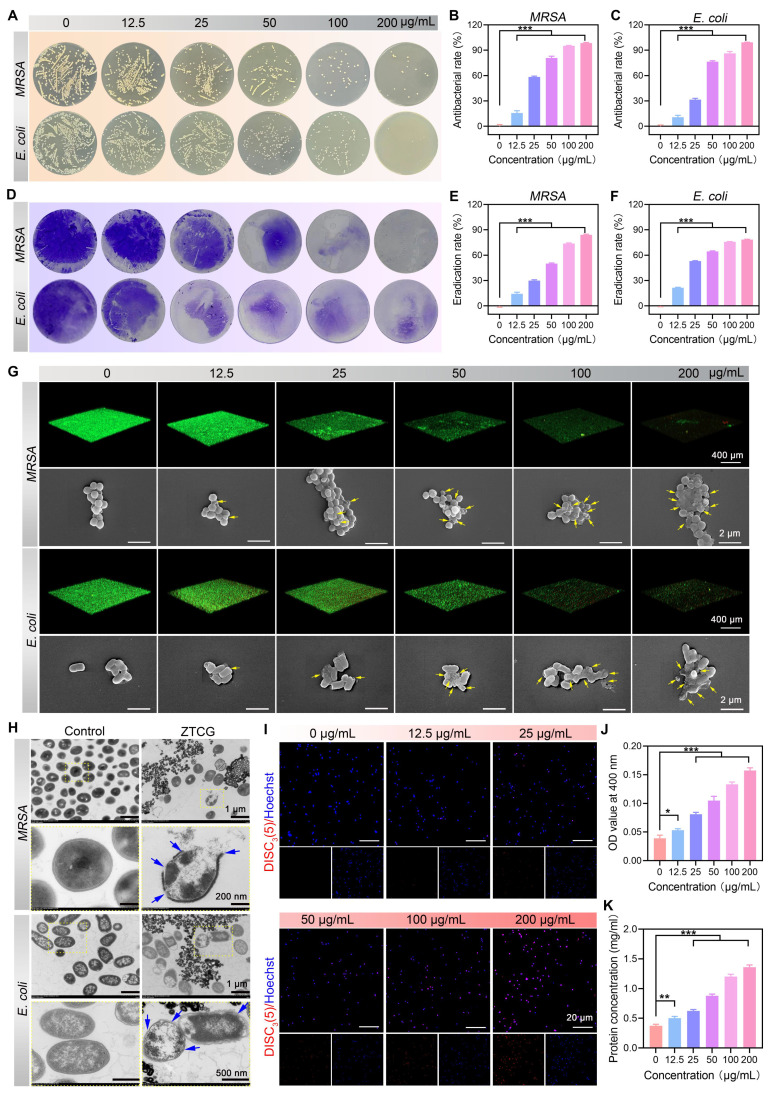
** Antibacterial and anti-biofilm activity of ZTCG nanozymes.** (A) Colony images of *MRSA* and* E. coli* after treatment with ZTCG nanozymes. (B, C) Antibacterial rates of ZTCG nanozymes against *MRSA* (B) and *E. coli* (C). (D) Crystal violet-stained images of bacterial biofilms (*MRSA* and *E. coli*) after treatment with ZTCG nanozymes. (E, F) Quantitative analysis of biofilm eradication. (G) Fluorescence staining images and SEM images of different bacterial biofilms after treatment with ZTCG nanozymes (yellow arrows indicate dead bacteria). (H) TEM images of *MRSA* and *E. coli* after treatment with PBS or ZTCG nanozymes (blue arrows indicate damaged sites of the bacterial cell membrane). (I) Bacterial membrane potential staining of *MRSA* after treatment with ZTCG nanozymes. (J) Quantitative analysis of membrane permeability. (K) Quantitative analysis of protein leakage. Values are expressed as the mean ± SD. (n = 3). *p < 0.05, **p < 0.01, ***p < 0.001.

Due to impaired immune function, diabetic wounds are particularly vulnerable to recurrent pathogenic bacterial infections and biofilm formation, which can lead to drug resistance and ultimately result in refractory wound healing 39. To evaluate the broad-spectrum antibacterial performance of the ZTCG nanozymes, *MRSA* and *E. coli* were used as model strains. The antibacterial activity was determined by the plate count method. As shown in **Figure 3A**, the number of bacterial colonies for both strains decreased significantly with increasing concentrations of the ZTCG nanozymes. At 200 μg/mL, the antibacterial rates against *MRSA* and *E. coli* reached 98.55 ± 0.71% and 99.37 ± 0.21%, respectively (Figure 3B, C). These results demonstrate that ZTCG nanozymes possess a highly effective, broad-spectrum antibacterial capability.

One important mechanism of bacterial drug resistance is the production of biofilms. Live/dead bacterial fluorescence staining and the crystal violet staining technique were used to further explore the anti-biofilm activity of ZTCG nanozymes *in vitro*. Crystal violet staining results showed that the biofilm structure remained intact at 0 μg/mL but became severely disrupted with increasing ZTCG nanozyme concentrations (Figure 3D). At 200 μg/mL, the biofilm eradication rates reached 84.14 ± 0.66% and 78.47 ± 0.44% for *MRSA* and *E. coli*, respectively (Figure 3E, F). CLSM observations were highly consistent with these results. Biofilms with a high density of live bacteria (green fluorescence) persisted at 0 μg/mL. Treatment with low concentrations of ZTCG nanozymes caused partial biofilm damage and an increase in dead bacteria (red fluorescence). When the concentration was increased to 100 μg/mL, the biofilms were completely destroyed, and nearly no live bacteria were observed in the field of view (Figure 3G). These results clearly demonstrate the exceptional performance of ZTCG nanozymes in eradicating biofilms. Further SEM analysis showed that as the ZTCG nanozymes concentration increased, bacterial cell membranes became visibly ruptured and shrunken, with evidence of intracellular content leakage (Figure 3G).

To better understand the antibacterial mechanism,* MRSA* was used as the model bacterium. TEM observations (Figure 3H) showed that untreated control bacteria maintained their structural integrity and exhibited uniformly distributed intracellular material. In contrast, after treatment with ZTCG nanozymes, bacterial membranes were severely damaged, accompanied by the outflow of cytoplasmic contents. These findings suggest that the antibacterial action might be related to bacterial membrane damage. This hypothesis was confirmed using the fluorescent probe DiSC_3_ to detect changes in bacterial membrane potential 40. The fluorescence intensity increased significantly with higher ZTCG nanozymes concentration, indicating that the nanozymes induced bacterial membrane depolarization (Figure 3I). Furthermore, the ONPG hydrolysis experiment was used to assess the bacterial membrane permeability 41. The amount of ONPG hydrolysis increased with rising ZTCG nanozymes concentrations (Figure 3J), confirming that the nanozymes enhance bacterial membrane permeability. Further evidence of reduced membrane integrity was provided by increased protein leakage in the bacterial culture supernatant (Figure 3K).

In summary, the antibacterial mechanism of the ZTCG nanozyme is primarily attributed to membrane disruption, leading to membrane depolarization and increased permeability, which consequently causes leakage of essential intracellular components, such as proteins, ultimately achieving a potent bactericidal effect.

### Fabrication and characterization of the bilayer microneedle and its H_2_O_2_ visual monitoring capability

**Figure 4:**
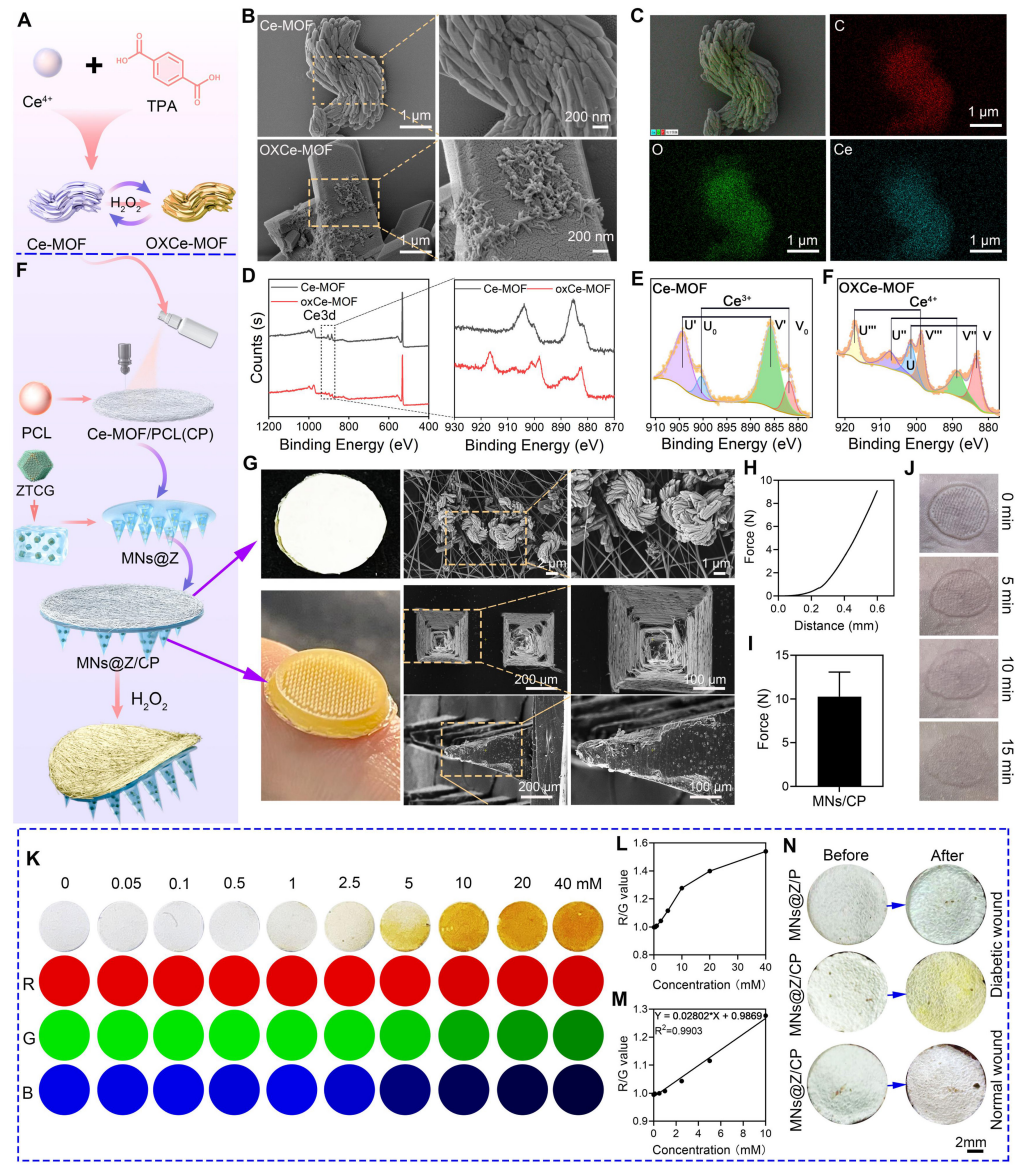
Physical characterization and H_2_O_2_-responsive colorimetric properties of MNs@Z/CP. (A) Schematic diagram of the preparation of Ce-MOF and its H_2_O_2_-responsive color transition. (B) SEM images of Ce-MOF and OXCe-MOF. (C) EDS images of Ce-MOF. (D) XPS spectra of Ce-MOF and OXCe-MOF. (E) Ce 3d XPS spectra of Ce-MOF and OXCe-MOF. (F) Schematic diagram illustrating the fabrication of MNs@Z/CP and its H_2_O_2_-triggered color transition. (G) Photographs and SEM images of the backing layer (CP electrospun membrane) and microneedle array. (H, I) Stress-displacement curve (H) and quantification (I) of MNs@Z/CP. (J) Photos of MNs@Z/CP penetrating rat skin at 0-15 min. (K) H_2_O_2_-responsive colorimetric transition of the CP electrospun membrane and corresponding quantitative RGB (Red, Green, Blue) analysis. (L, M) R/G (red/green) value and H_2_O_2_ concentration curve (L) and linear correlation analysis (M). (N) Color changes of MNs@Z/CP before and after application on diabetic and normal wound.

A cerium-based MOF (Ce-MOF) was synthesized by adopting a previously reported approach with optimization (**Figure 4A**) 27. SEM results showed that the pristine Ce-MOF exhibited a flower-like structure, while after being treated with H_2_O_2_, the OXCe-MOF transformed into a rod-like structure (Figure 4B). EDS analysis confirmed that Ce, O, and C elements were uniformly distributed in Ce-MOF (Figure 4C). High-resolution XPS spectra revealed that Ce in the pristine Ce-MOF existed primarily in the Ce^3+^ state. Following H_2_O_2_ treatment, the Ce^4+^/Ce^3+^ ratio in OXCe-MOF significantly increased (Figure 4D, E), and its color turned yellow (Figure S5), suggesting a strong colorimetric response of Ce-MOF to H_2_O_2_. We evaluated the selectivity of the colorimetric response of Ce-MOF against a panel of potential interferents commonly present in the wound microenvironment. As shown in Figure S6, the Ce-MOF exhibited a distinct and significant colorimetric response only in the presence of H_2_O_2_. In contrast, no notable color change was observed upon exposure to water, acidic conditions (pH 5.5), potassium chloride (KCl), sodium chloride (NaCl), glucose, uric acid (UA), ascorbic acid (AA), or L-lysine. These results confirm the high selectivity of the Ce-MOF sensing layer toward H_2_O_2_, supporting its suitability for specific oxidative stress monitoring in complex wound exudate environments.

The bilayer MNs@Z/CP patch was fabricated via a template casting method. The needle layer consisted of a HAMA hydrogel loaded with ZTCG nanozymes (MNs@Z) for antibacterial and ROS-scavenging purposes. The backing layer was made of an electrospun PCL membrane spray-coated with Ce-MOF (CP layer), which served as the colorimetric component (Figure 4F). Macroscopically, the patch exhibited a bilayer structure consisting of a well-defined brown microneedle layer and a white backing layer (Figure 4G). Cross-sectional SEM views of the needle indicated a height of 600 μm and a base diameter of 360 μm (Figure 4G). The electrospun fiber surface displayed uniformly distributed Ce-MOF particles, as verified by SEM and EDS (Figure 4G and Figure S7). Mechanical compression tests showed that a force of 10.29 ± 2.79 N was required at a compression displacement of 0.6 mm (Figure 4H, I), demonstrating sufficient mechanical strength for skin insertion without fracture 42. The minimally invasive nature was demonstrated by *in situ* implantation tests on a rat skin model, which revealed full epidermal penetration with distinct needle holes after insertion, followed by progressive tissue healing and pore closure (Figure 4J). According to the drug release experiment shown in Figure S8, Ga^3+^ and Cu^2+^ were continuously released from the microneedles, thereby achieving the therapeutic effect.

When Ce^3+^ is oxidized to Ce^4^⁺, the color of Ce-MOF transitions from white to yellow, and the color intensity is positively correlated with the concentration of H_2_O_2_ 27. CP films were immersed in H_2_O_2_ solutions at different concentrations to measure this response and ImageJ software was used to extract RGB values from optical images (Figure 4K). The regression equation R/G = 0.02802 × H_2_O_2_ (mmol/L) + 0.9869 describes the good linear relationship between H_2_O_2_ concentration and the R/G value, which allowed for the creation of a standard color chart (Figure 4L, M). Using this equation and color chart, semi-quantitative assessment of wound H_2_O_2_ levels becomes feasible. In animal studies, application of MNs@Z/CP to diabetic wounds induced a visible color change (Figure 4N), whereas the control group without Ce-MOF coating showed no such response. Moreover, no color change was observed when MNs@Z/CP was applied to normal wounds. This color change is attributed to the elevated H_2_O_2_ levels in the diabetic wound microenvironment resulting from inflammation and hyperglycemia. These findings show that MNs@Z/CP can visually monitor wound H_2_O_2_. Based on the color change of MNs@Z/CP, the next steps in the treatment plan, such as whether to replace the patch, can be determined. Compared with conventional nanozyme microneedles that are limited to a single therapeutic function 43-45, the key advantage of MNs@Z/CP lies in its dual-function capability for both treatment and monitoring, enabling precise adjustment of therapeutic strategies based on real-time monitoring outcomes. Before treatment and on days 3 and 5 after MNs@Z/CP treatment, we measured the H_2_O_2_ concentration in wounds of diabetic rats using a colorimetric method. As shown in Figure S9, the color of the MNs@Z/CP microneedles gradually faded on days 3 and 5 post-treatment, and the corresponding mean RGB values were obtained according to the established relationship. The results indicated that due to MNs@Z/CP treatment, wound inflammation and oxidative stress were alleviated, leading to a gradual decrease in H_2_O_2_ concentration. Subsequently, we further measured the wound H_2_O_2_ levels of rats using an H_2_O_2_ assay kit, and the results were highly consistent with those obtained from the microneedle monitoring (Figure S10). Collectively, these findings demonstrate that the microneedles can effectively monitor wound H_2_O_2_ concentration via colorimetric analysis.

A fundamental requirement for biomedical applications is excellent biocompatibility 15. Initially, we evaluated the biocompatibility of the ZTCG nanozymes through hemolysis assay, CCK-8 assay, and live/dead staining. Compared with ZIF-8, the MPN-coated ZTCG nanozymes exhibited improved biocompatibility (Figure S11). CCK-8 assays validated that ZTCG nanozymes were significantly less cytotoxic than ZIF-8 at 200 μg/mL (Figure S12). Furthermore, the hemolysis rate of ZTCG nanozymes was below 5% at concentrations up to 200 μg/mL but exceeded this threshold at 400 μg/mL (Figure S13). Consequently, the 200 μg/mL concentration was selected for microneedle preparation.

We further evaluated the cytotoxicity of the fabricated materials (MNs/P, MNs@Z/P, and MNs@Z/CP) towards HUVECs and L929 cells. After co-culture, cell viability of all material groups remained above 90% (Figure S14), suggesting high biosafety. Live/dead staining provided additional evidence for this conclusion (Figure S15). Moreover, the hemolysis experiment verified that all bilayer microneedles exhibited a hemolysis rate below 5% (Figure S16). While considerable breakage was seen in the positive group, microneedle-treated erythrocytes retained structural integrity, similar to the PBS group (Figure S16). Collectively, these findings demonstrate that MNs@Z/CP possesses outstanding biocompatibility, offering a strong basis for its *in vivo* application.

We also evaluated the anti-inflammatory and antibacterial properties of MNs@Z/CP *in vitro*. Owing to the addition of ZTCG nanozymes, both MNs@Z/P and MNs@Z/CP had improved ABTS^•^ and DPPH^•^ scavenging capabilities, with MNs@Z/CP demonstrating more potent free radical elimination capability (Figure S17). At the same time, MNs@Z/P and MNs@Z/CP showed strong antibacterial activity against *MRSA* and *E. coli* (Figure S18). Notably, MNs@Z/CP demonstrated higher anti-inflammatory and antibacterial efficacy than MNs@Z/P, which can be attributed to the synergistic effect of the integrated Ce-MOF's inherent antibacterial properties and radical scavenging capacity.

### MNs@Z/CP promotes cell proliferation, migration, and angiogenesis by alleviating oxidative stress *in vitro*

Non-enzymatic protein glycation leads to considerable accumulation of advanced glycation end products (AGEs) in diabetes. By triggering the AGEs-RAGE signaling pathway, AGEs not only directly induce oxidative stress but also synergistically elevate intracellular ROS levels 46. These changes result in a persistently inflamed wound microenvironment, severely impeding the healing process. We further assessed the capacity of the MNs@Z/CP bilayer microneedles to scavenge ROS, reduce oxidative stress, and preserve cell viability *in vitro*.

**Figure 5:**
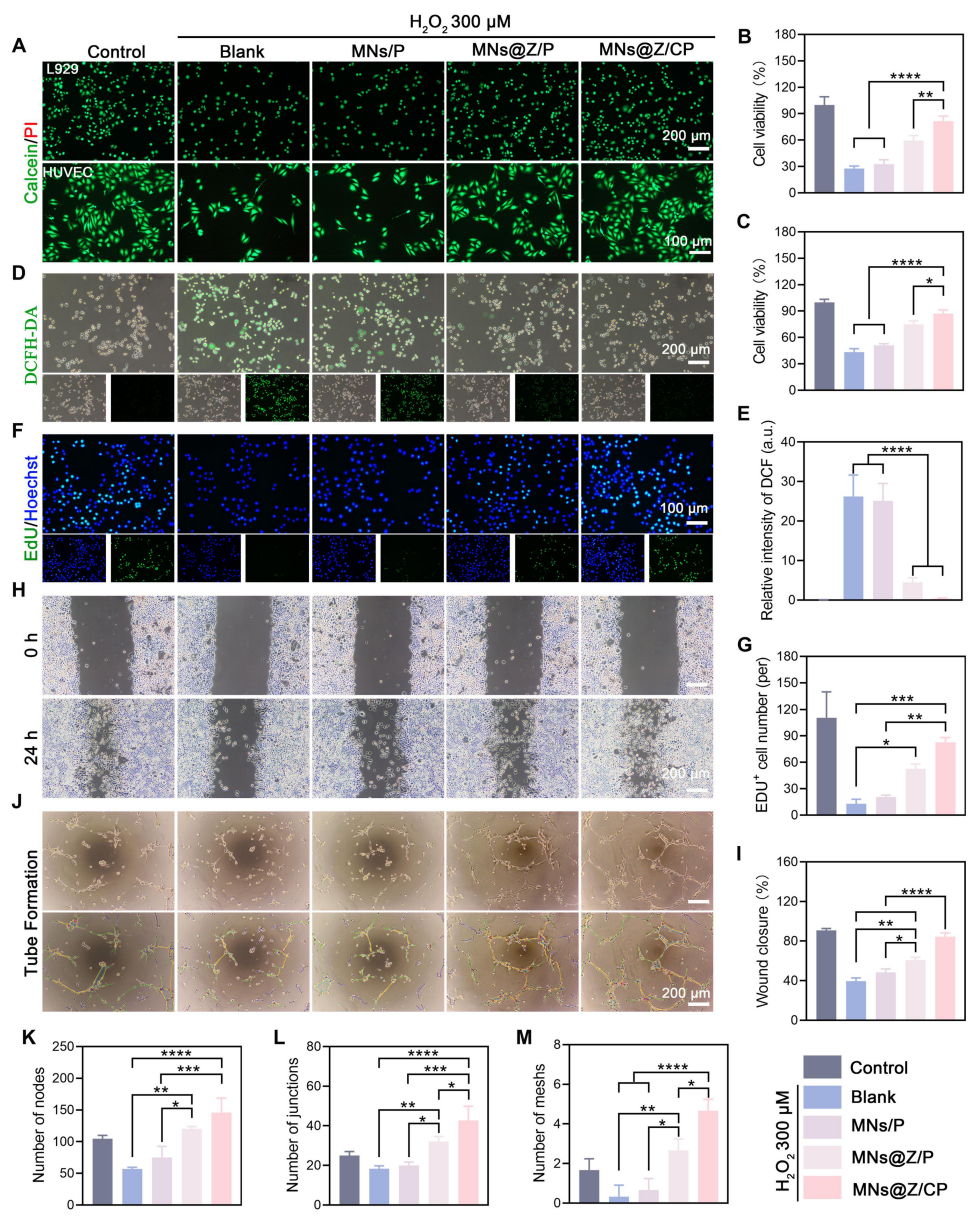
MNs@Z/CP promotes cell proliferation, migration, and tube formation under inflammatory conditions *in vitro*. (A) Live/dead staining images after different treatments. (B, C) Cell viability of L929 cells (B) and HUVECs (C) after different treatments. (D, E) Scavenging capacity of MNs@Z/CP against H_2_O_2_-induced ROS assessed by DCFH-DA staining (D) and its quantitative analysis (E). (F, G) Immunofluorescence images of EdU staining (F) and its quantification (G) in L929 cells (n = 5). (H) Images illustrating scratch wound healing (n = 3). (I) Quantification of the scratch assay (n = 3). (J) Tube formation images after different treatments. (K-M) Quantification of the number of nodes (K), junctions (L), and meshes (M) (n = 3). Values are expressed as the mean ± SD. *p < 0.05, **p < 0.01, ***p<0.001.

First, we investigated the cytoprotective effect of MNs@Z/CP against H_2_O_2_-induced oxidative stress. Live/dead cell labeling and CCK-8 tests showed that H_2_O_2_ treatment dramatically decreased the viability of both L929 cells and HUVECs. In contrast, treatment with either MNs@Z/P or MNs@Z/CP effectively reversed this trend, significantly improving cell survival under oxidative stress (**Figure 5A-C**). DCFH-DA staining was used to measure intracellular ROS levels. H_2_O_2_ treatment markedly increased intracellular ROS, as seen in Figure 5D and E, whereas both MNs@Z/P and MNs@Z/CP treatments reduced this H_2_O_2_-induced ROS elevation. EdU assays further demonstrated that MNs@Z/CP effectively maintained cell proliferation activity after H_2_O_2_ exposure (Figure 5F, G). These findings confirm the excellent ROS-scavenging ability of MNs@Z/CP and its corresponding cytoprotective function.

Coordinated cellular processes, such as fibroblast-driven granulation and endothelial-mediated angiogenesis, are necessary for diabetic wound healing. The persistent oxidative stress frequently interferes with these processes 47. We further evaluated the effects of MNs@Z/CP on cell migration and angiogenesis under oxidative stress conditions. In the scratch assay, fibroblast migration was impaired in the H_2_O_2_ group, whereas MNs@Z/CP significantly enhanced fibroblast migration, achieving an 84.43 ± 3.78% wound closure rate (Figure 5H, I). Tube formation assays demonstrated that MNs@Z/CP reversed the H_2_O_2_-induced disruption of tubular structures, leading to the formation of a dense and interconnected tube network (Figure 5J). Quantitative analysis further confirmed the superior pro-angiogenic activity of MNs@Z/CP under oxidative stress conditions (Figure 5K–M). This study demonstrates that MNs@Z/CP effectively protects cells from oxidative damage by scavenging ROS. As a result, it increases the angiogenic capacity of endothelial cells, promotes fibroblast migration and proliferation under inflammatory conditions, and accelerates diabetic wound healing.

### MNs@Z/CP induces macrophage reprogramming by activating the Nrf2/HO-1 pathway

**Figure 6:**
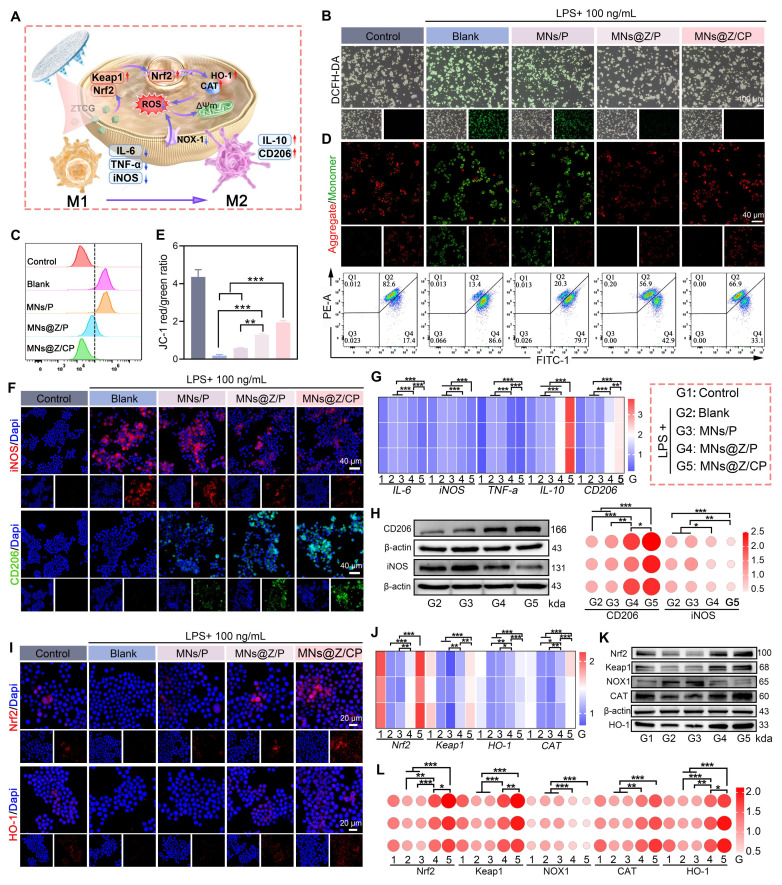
MNs@Z/CP reprograms macrophages via the Nrf2/HO-1 pathway *in vitro*. (A) Schematic diagram elucidating the proposed mechanism of macrophage reprogramming. (B) DCFH-DA staining images. (C) Flow cytometry of intracellular ROS. (D) ΔΨ_m_ assessment using JC-1 staining and flow cytometry analysis. (E) Quantitative analysis of JC-1 staining. (F) Immunofluorescence images of iNOS and CD206 in macrophages. (G) RT-qPCR analysis of mRNA levels for *IL-6*, *TNF-α*, *iNOS*, *IL-10*, and *CD206*. (H) Western blot of CD206 and iNOS protein levels and quantitative bubble plot. (I) Fluorescence images of Nrf2 and HO-1 in macrophages. (J) RT-qPCR analysis of mRNA levels for *Nrf2*, *Keap1*, *HO-1*, and *CAT*. (K) Western blot analysis of Nrf2, Keap1, NOX1, HO-1, and CAT. (L) Corresponding quantitative bubble plot. Values are expressed as the mean ± SD. (n = 3). *p < 0.05, **p < 0.01, ***p < 0.001.

We further investigated the underlying antioxidant mechanisms of MNs@Z/CP at the cellular level (**Figure 6A**). DCFH-DA fluorescence images showed that LPS stimulation significantly increased green fluorescence in macrophages, indicating elevated ROS levels (Figure 6B). Treatment with MNs@Z/P and MNs@Z/CP effectively scavenged intracellular ROS, resulting in a marked reduction in green fluorescence. Flow cytometry quantification confirmed that ROS levels in the MNs@Z/CP group were significantly lower than in all other treatment groups (Figure 6C).

Given the critical role of ROS in mitochondrial function, we subsequently measured changes in ΔΨ_m_ using JC-1 staining (Figure 6D). The findings demonstrated that LPS treatment reduced ΔΨ_m_, indicating compromised mitochondrial activity. In contrast, both MNs@Z/P and MNs@Z/CP significantly restored ΔΨ_m_, with the MNs@Z/CP group showing the strongest effect. Quantitative flow cytometry results (Figure 6E) further verified that the treatment effectively mitigated LPS-induced mitochondrial dysfunction.

Macrophage polarization is directly linked to the healing process of diabetic ulcers 48, 49. We subsequently explored whether MNs@Z/CP could remodel immune homeostasis by reprogramming macrophages. Theoretically, reducing intracellular ROS levels facilitates the polarization of macrophages towards the M2 phenotype. Immunofluorescence staining (Figure 6F) showed that MNs@Z/P and MNs@Z/CP treatments significantly upregulated CD206 (M2 marker, green fluorescence) expression, while suppressing iNOS (M1 marker, red fluorescence). RT-qPCR results (Figure 6G) indicated that MNs@Z/P and MNs@Z/CP effectively counteracted the LPS-induced upregulation of pro-inflammatory genes (*IL-6*, *iNOS*, *TNF-α*) and concurrently promoted the expression of M2-associated anti-inflammatory mediators (*IL-10*,* CD206*). WB analysis (Figure 6H) further demonstrated that the MNs@Z/P and MNs@Z/CP treatments shifted macrophage polarization toward the M2 phenotype. Notably, MNs@Z/CP exhibited superior cytoprotective, ROS scavenging, and M2-polarizing capabilities compared to MNs@Z/P. The superior performance of MNs@Z/CP may be ascribed to the combined action of Ce-MOF and ZTCG nanozymes, which synergistically improve therapeutic efficacy. In addition to the intrinsic activity of ZTCG, Ce-MOF utilizes the variable valence states of cerium ions (Ce^3+^/Ce^4+^) to scavenging free radicals via electron transfer interactions 50, thereby further alleviating oxidative stress and promoting microenvironmental remodeling.

The Nrf2/HO-1 signaling axis is essential for reducing oxidative stress, wherein Nrf2 exerts cytoprotective antioxidant effects mainly by upregulating the downstream effector HO-1 51, 52. We further investigated the impact of MNs@Z/CP on the Nrf2/HO-1 pathway to elucidate its anti-inflammatory mechanism. Immunofluorescence analysis revealed that LPS treatment downregulated Nrf2 and HO-1 expression, whereas MNs@Z/CP treatment significantly enhanced the intracellular intensity of both Nrf2 and HO-1 (Figure 6I). Based on these findings, we hypothesized that MNs@Z/CP may promote macrophage reprogramming by activating the Nrf2/HO-1 pathway. To validate this process, we conducted further mechanistic studies (Figure 6J-L). RT-qPCR results demonstrated that LPS suppressed the expression of *Keap1*, *Nrf2*, *HO-1*, and *CAT* in macrophages, while MNs@Z/CP treatment upregulated the expression of these genes (Figure 6J). Consistent WB data also showed that MNs@Z/CP therapy decreased the protein level of NOX1, a significant ROS producer. We speculate that this decrease in NOX1 protein levels results from macrophage polarization toward the M2 phenotype, which lowers inflammation and subsequently suppresses NOX1 production via feedback mechanism. Downregulation of NOX1 would further decrease intracellular ROS levels, establishing a positive feedback regulatory loop (Figure 6K, L).

In summary, MNs@Z/CP alleviates mitochondrial dysfunction and reprograms macrophages toward the M2 phenotype by activating the intrinsic Keap1/Nrf2/HO-1 antioxidant cascade, thereby creating a microenvironment conducive to tissue healing.

### MNs@Z/CP promotes the healing of diabetic wounds

**Figure 7:**
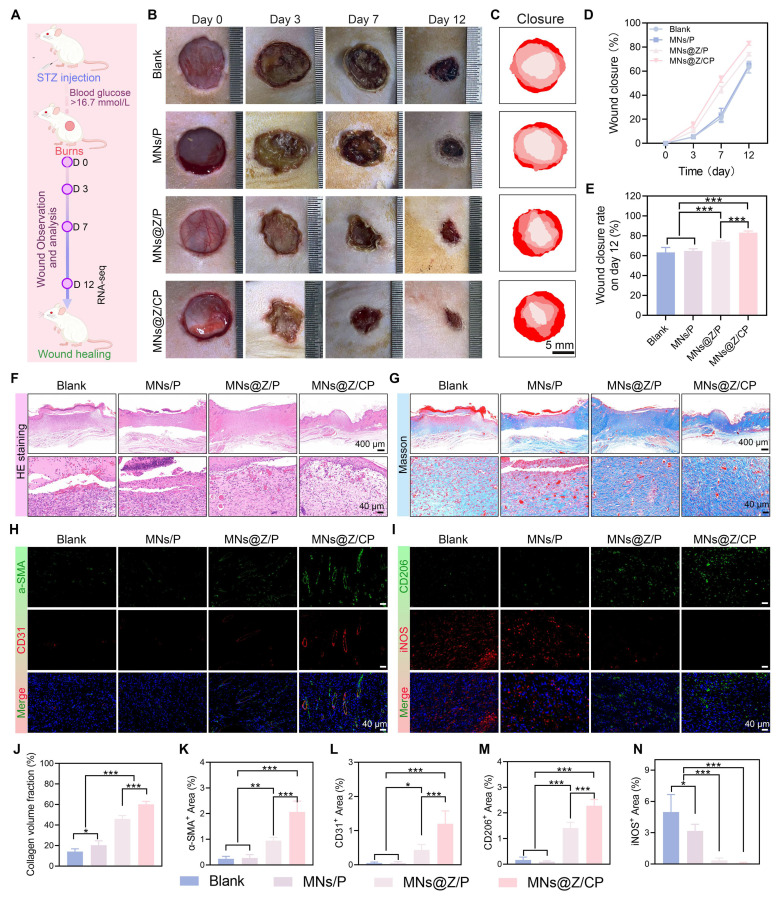
** Therapeutic efficacy of MNs@Z/CP in diabetic wounds.** (A) Experimental schematic of establishing diabetic wounds. (B) Representative photographs of wound healing process in the blank, MNs/P, MNs@Z/P, and MNs@Z/CP groups. (C) Wound traces in each group. (D) Wound closure rate curve. (E) Wound closure rate on day 12. (F, G) H&E (F) and Masson (G) staining images. (H) Immunofluorescence staining images for iNOS and CD206 of wound tissues. (I) Immunofluorescence staining images for α-SMA and CD31 of wound tissues. (J) Quantitative analysis of collagen fraction. (K-N) Quantified areas positive for α-SMA (K), CD31 (L), CD206 (M), and iNOS (N). Values are expressed as the mean ± SD. (n = 5). *p < 0.05, **p < 0.01, ***p < 0.001.

To systematically evaluate the therapeutic performance of the MNs@Z/CP patch, a STZ-induced diabetic rat model was used (**Figure 7A**) 4. Wounds were observed and captured on days 0, 3, 7, and 12 post-treatments (Figure 7B, C). Compared with the blank and MNs/P groups, accelerated wound contraction was evident in the MNs@Z/P and MNs@Z/CP groups as soon as day 3. Notably, the MNs@Z/CP group showed a wound closure rate of 53.32 ± 2.73% on day 7, higher than that of any other group. By day 12, the MNs@Z/P group exhibited a closure rate of 74.15 ± 1.21%, whereas the blank and MNs/P groups still had substantial unhealed wound areas (closure rates of 63.34 ± 4.89% and 64.84 ± 2.07%, respectively). In contrast, the MNs@Z/CP group achieved a closure rate of 83.17 ± 1.66% on day 12, representing the most complete wound closure (Figure 7D, E).

On day 12, we harvested the wound sites and adjacent skin tissues for histological assessment. H&E staining (Figure 7F) indicated that the wound in the MNs@Z/CP group was almost entirely covered by epithelial tissue, with intact epidermal regeneration and densely packed granulation tissue. In contrast, both the blank and the MNs/P groups still exhibited scab formation and inflammatory cell infiltration. Granulation tissue formed in the MNs@Z/P group but remained immature, and epidermal regeneration was insufficient. These results demonstrate that MNs@Z/CP therapy successfully reduced inflammation and promoted wound healing.

### MNs@Z/CP promotes collagen deposition, angiogenesis, and macrophage reprogramming in diabetic wounds

Optimal wound healing quality is critically dependent upon adequate collagen deposition, which provides essential support for tissue remodeling, cell proliferation, and differentiation 53. Masson's trichrome staining (Figure 7G, J) demonstrated that the MNs@Z/CP group exhibited the most abundant and well-organized collagen deposition. CD31 is a marker of nascent endothelial cells during early angiogenesis, while α-SMA indicates vascular maturity and pericyte recruitment 54, 55. Therefore, angiogenesis was assessed by immunofluorescence staining for CD31 and α-SMA. The blank group displayed only weak positive signals (Figure 7H). Within the wound region, CD31 and α-SMA expression were markedly increased in the MNs@Z/P group and were most intense in the MNs@Z/CP group, indicating the superior pro-angiogenic capacity of the MNs@Z/CP group (Figure 7K, L).

During wound healing, macrophages are essential for immune regulation 56. The delayed M1 to M2 transition of macrophages impairs tissue regeneration in diabetic wounds. Using immunofluorescence labeling, we evaluated the expression of iNOS and CD206. As illustrated in Figure 7I, MNs@Z/CP treatment significantly decreased the iNOS-positive area while increasing the CD206-positive area compared with the blank group. Specifically, the MNs@Z/CP group showed the lowest iNOS expression and the highest level of CD206 expression (Figure 7M, N), suggesting that this therapy successfully promoted macrophage reprogramming toward the M2 phenotype.

In summary, the bilayer MNs@Z/CP patch promotes macrophage phenotypic shift, enhances collagen deposition and angiogenesis, synergistically inhibits the inflammatory response, and accelerates tissue remodeling to enable rapid and high-quality healing of diabetic wounds.

### MNs@Z/CP targets the IL-17 signaling pathway and cell cycle to remodel the diabetic wound microenvironment

**Figure 8:**
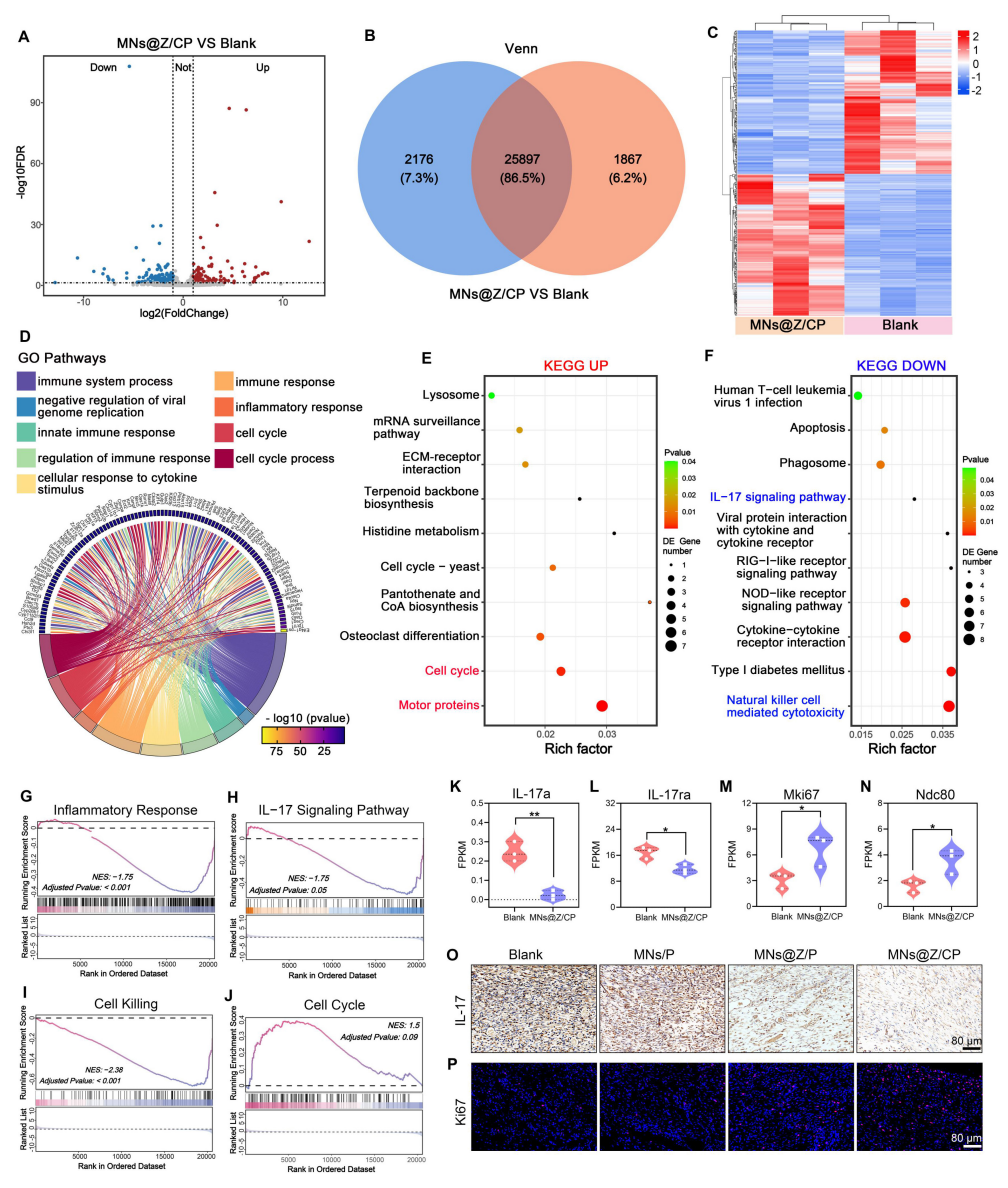
** Mechanistic analysis of MNs@Z/CP-mediated promotion of diabetic wound healing.** (A) Volcano plot of DEGs between the MNs@Z/CP and blank groups. (B) Venn diagram of DEGs between the MNs@Z/CP and blank groups. (C) Clustering heatmap of DEGs. (D) GO enrichment analysis. (E, F) KEGG pathway analysis for up-regulated (E) and down-regulated (F) DEGs. (G-J) GSEA plot for the inflammatory response, IL-17 signaling pathway, cell killing and cell cycle pathway. (K-N) Fragments per kilobase million (FPKM) values of *IL-17a*, *IL-17ra*, *Mki67* and *Ndc80* (n = 3). (O) Immunohistochemical staining of IL-17 on day 12. (P) Immunofluorescence staining of Ki67 on day 12. Values are expressed as the mean ± SD. *p < 0.05, **p < 0.01.

Although MNs@Z/CP has shown promising therapeutic outcomes, a comprehensive understanding of its anti-inflammatory and tissue remodeling mechanisms is lacking. We performed transcriptomic sequencing analysis on rat wound tissues from the MNs@Z/CP-treated and blank groups 12 days post-treatment. The volcano plot illustrated significant differential gene expression (**Figure 8A**), and Venn analysis identified 4043 differentially expressed genes (DEGs) (Figure 8B). Unsupervised clustering analysis indicated clear separation between the two groups (Figure 8C), suggesting that MNs@Z/CP exerts a global regulatory effect on the wound healing transcriptome.

To characterize the biological processes associated with these DEGs, we performed KEGG and GO enrichment analysis. GO analysis showed that the DEGs were predominantly enriched in immune response regulation and cell cycle-related processes (Figure 8D), providing a molecular basis for the immune modulation and repair promotion mediated by MNs@Z/CP. KEGG pathway analysis further showed that MNs@Z/CP treatment was strongly associated with activation of the cell cycle pathway and downregulation of the IL-17 signaling pathway and natural killer cell-mediated cytotoxicity pathway (Figure 8E, F). Previous studies have shown that ROS activates the IL-17 pathway, triggering a pro-inflammatory cascade that releases TNF-α, CCL2, and IL-1β 57, 58. This leads to M1 macrophage polarization and neutrophil recruitment and activation, thereby exacerbating local inflammation. These factors can worsen oxidative stress, inhibit cell division, and cause cell death, ultimately impairing diabetic wound healing 9, 59. Herein, MNs@Z/CP effectively suppressed the IL-17 signaling pathway, thereby reducing inflammation, promoting macrophage transition toward the M2 phenotype, and establishing a pro-regenerative cytokine milieu that synergizes with cell cycle progression to accelerate wound healing.

To validate these enrichment analysis results, we used gene set enrichment analysis (GSEA) on the "immune response" (GO), "IL-17 signaling pathway" (KEGG), "cell killing" (GO), and "Cell Cycle" (KEGG) pathways (Figure 8G-J). GSEA results indicated an overall suppression of the immune response and the IL-17 pathway in the treated group (Figure 8H). This finding is significant because the IL-17 cytokine, which is chronically overexpressed in diabetic wounds, exacerbates tissue damage by inducing matrix metalloproteinases that degrade the extracellular matrix and disrupt cell-matrix adhesion 60. Our data confirmed the downregulation of specific pathway components, including *IL1R1*, *IL17RA*, *IL17A*, and the downstream genes *FOSL1* and *S100A9* (Figure 8K, L and Figure S19). Concurrently, GSEA revealed that MNs@Z/CP inhibited cell killing-related pathways while promoting cell cycle progression. Ki-67, a nuclear protein widely used as a marker for active cell cycle phases, directly reflects tissue proliferative status 61. In this study, Ki67 expression was significantly upregulated in the MNs@Z/CP-treated group, as were mitosis-associated genes such as *NDC80*, *SGO*1, and *KNL1* (Figure 8M, N and Figure S20). Furthermore, immunohistochemical staining for IL-17 and immunofluorescence staining for Ki67 further confirmed these findings. The blank group still exhibited high levels of IL-17 expression on day 12, indicating persistent inflammation (Figure 8O and Figure S21). In contrast, MNs@Z/CP treatment significantly reduced IL-17 levels, with the MNs@Z/P group also showing a moderate decrease, demonstrating the clear anti-inflammatory effect conferred by the incorporation of ZTCG. Moreover, the MNs@Z/CP-treated group showed markedly higher Ki67 fluorescence signal in wound regions, indicating enhanced cellular proliferative activity (Figure 8P and Figure S22).

Remarkably, the MNs@Z/CP-treated group showed higher expression of antioxidant enzyme-related genes, including *CAT* and *GPX7* (Figure S23), consistent with our *in vitro* findings. Collectively, these results demonstrate that MNs@Z/CP increases the production of antioxidant enzymes *in vivo*, scavenges ROS to alleviate oxidative stress, and remodels the wound microenvironment while blocking the IL-17-mediated inflammatory response. This promotes cell cycle progression and proliferation, exerting potent anti-inflammatory and pro-repair effects, and provides a crucial molecular basis for diabetic wound healing.

### MNs@Z/CP promotes healing of *MRSA*-infected diabetic wounds through multi-dimensional mechanisms

**Figure 9:**
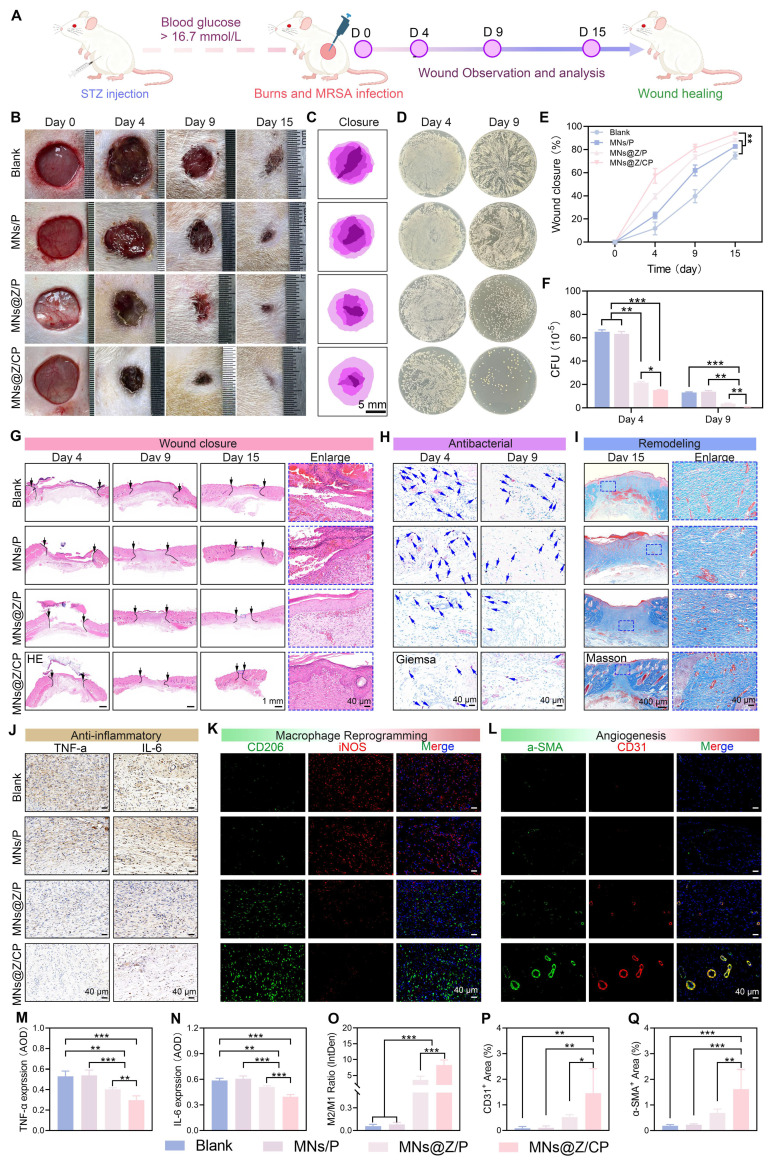
** Therapeutic efficacy of MNs@Z/CP in *MRSA*-infected diabetic wounds.** (A) Experimental schematic of the wound model established. (B) Representative photographs of wound healing process in the blank, MNs/P, MNs@Z/P, and MNs@Z/CP groups. (C) Wound traces in each group on day 15. (D) Bacterial colony counts from wounds on day 4 and day 9 post-treatment. (E) Wound closure rate. (F) Quantitative evaluation of bacterial colonies from wounds. (G-I) H&E (G), Giemsa (H), and Masson (I) staining images (black arrows: wound area; blue arrows: bacteria). (J) Immunohistochemical staining images for TNF-α and IL-6 in wound tissues. (K) Immunofluorescence staining images for iNOS and CD206 in wound tissues. (L) Immunofluorescence staining images for α-SMA and CD31 in wound tissues. (M, N) Quantitative analysis of TNF-α (M) and IL-6 (N). (O) Ratio of M2 (CD206⁺) / M1 (iNOS⁺). (P, Q) Quantified areas positive for CD31 (P) and α-SMA (Q). Values are expressed as the mean ± SD. (n = 5). *p < 0.05, **p < 0.01, ***p < 0.001.

The hyperglycemic microenvironment in diabetic wounds facilitates secondary infections, and the clinical overuse of antibiotics exacerbates bacterial resistance 62, 63. To evaluate the therapeutic potential of MNs@Z/CP against drug-resistant bacterial wound infections, we established a full-thickness *MRSA*-infected wound model on diabetic rats and applied the microneedles for treatment (**Figure 9A**).

Wound appearances on days 0, 4, 9, and 15 for each treatment group are shown in Figures 9B and 9C. After 15 days of therapy, the MNs@Z/CP group exhibited markedly reduced wound area and abscess size compared with the blank group (Figure 9E). Agar plate colony counting results (Figure 9D, F) revealed that MNs@Z/P treatment achieved an antibacterial rate of 73.38 ± 2.21%, and the MNs@Z/CP group further increased this rate to 94.88 ± 1.04% on day 9. This enhanced antibacterial effect may arise from the inherent antibacterial properties of the Ce-MOF component, which has been reported to possess potent bacteriostatic activity. Furthermore, no obvious pathological damage to the major organs of rats was observed after 15 days of treatment, demonstrating the system's superior *in vivo* biosafety and therapeutic potential (Figure S24).

H&E staining results (Figure 9G) showed that the blank and MNs/P groups displayed poor epidermal integrity, large wound areas, and substantial inflammatory cell infiltration. In contrast, the MNs@Z/P and MNs@Z/CP groups exhibited much smaller wound areas and significantly reduced inflammatory cell infiltration, with the MNs@Z/CP group showing the most pronounced benefit. Giemsa staining further indicated that the MNs@Z/P and MNs@Z/CP groups also had significantly fewer residual bacteria (blue arrows) than the blank and MNs/P groups 64, with the MNs@Z/CP group achieving the most thorough bacterial clearance (Figure 9H and Figure S25). Masson's trichrome staining (Figure 9I) further showed that the MNs@Z/CP group exhibited the highest collagen deposition. Effective collagen deposition is essential during wound repair, and its increased content accelerates tissue regeneration. These results demonstrate that MNs@Z/CP can efficiently reduce inflammation, promote collagen production, and rebuild skin structure, thereby facilitating the healing of *MRSA-*infected diabetic wounds.

To further examine the anti-inflammatory effects of MNs@Z/CP and its regulatory capacity on macrophage polarization during the infected wound repair process, we performed immunohistochemical analyses on day 9. Immunohistochemical labeling revealed markedly higher expression levels of TNF-α and IL-6 in the blank and MNs/P groups than the other groups (Figure 9J, M, and N), suggesting a robust inflammatory response. In the MNs@Z/P group, TNF-α and IL-6 expression dropped, reflecting reduced inflammatory response due to the multi-enzyme activity, radical scavenging capacity of ZTCG nanozymes, and TA release. The MNs@Z/CP group further decreased the expression of these pro-inflammatory cytokines to the lowest level, owing to the synergistic antioxidant effect of the Ce-MOF nanoparticles, highlighting its superior anti-inflammatory performance.

Macrophage phenotypes were assessed using double immunofluorescence labeling for iNOS (red, M1) and CD206 (green, M2). The blank and MNs/P groups exhibited strong red fluorescence and weak green fluorescence, whereas the MNs@Z/CP group showed the opposite pattern (Figure 9K). Quantitative regional analysis (Figure 9O) indicated that both the MNs@Z/P (3.61 ± 1.17) and MNs@Z/CP (8.25 ± 1.59) groups exhibited higher M2/M1 macrophage ratios than the blank group (0.06 ± 0.02). The MNs@Z/CP group showed the greatest elevation, demonstrating its ability to effectively promote M2-type macrophage polarization. Additionally, CD31 immunofluorescence staining showed that the MNs@Z/P and MNs@Z/CP groups had considerably larger CD31-positive vascular areas than the blank group, indicating higher capillary density and active angiogenesis (Figure 9L, 9P). The α-SMA-positive area also increased in these two groups, suggesting that ZTCG nanozymes support vascular maturation (Figure 9L). Notably, the MNs@Z/CP group exhibited a larger α-SMA-positive area than the MNs@Z/P group (Figure 9Q). This may be attributed to the greater capacity of the MNs@Z/CP group to polarize macrophages toward the M2 phenotype, which in turn promotes the generation of more pro-angiogenic cytokines 65.

In summary, MNs@Z/CP effectively eliminates deep bacterial infections and reduces the inflammatory cascade through its multiple antioxidant actions. By remodeling the local immune microenvironment, it promotes the transition of macrophages to M2 phenotype, which subsequently stimulates the expression of pro-angiogenic factors, accelerates tissue regeneration and structural reconstruction, and ultimately enables rapid and excellent healing of *MRSA*-infected diabetic wounds.

## Conclusion

In conclusion, this work successfully established an intelligent bilayer microneedle system based on the integration of dual MOFs, establishing a novel paradigm of integrated therapy and monitoring for chronic wounds. This system effectively clears drug-resistant bacterial biofilms, remodels the oxidative stress microenvironment, modulates immune responses, and enables real-time monitoring of wound H_2_O_2_ levels. Mechanistically, the system alleviates oxidative damage by triggering the Nrf2/HO-1 pathway and promotes macrophage reprogramming to M2 phenotype *in vitro*. It mitigates the inflammatory response by downregulating the IL-17 signaling pathway, synergistically promoting angiogenesis and collagen remodeling *in vivo*. These effects establish a comprehensive therapeutic framework encompassing anti-inflammatory, immunomodulatory, pro-angiogenic, and pro-proliferative mechanisms. This multi-layered spatial regulation strategy not only successfully addresses the challenges of intractable infection, oxidative stress imbalance, and inflammatory dysregulation in chronic wound healing, but also achieves on-demand precision therapy. This bilayer microneedle system effectively breaks the cycle of chronic inflammation by integrating monitoring functions with therapeutic interventions, laying the foundation for the next-generation smart wound management. Its potential applications extend to various refractory wounds, such as diabetic ulcers and drug-resistant bacterial infections.

## Supplementary Material

Supplementary figures and tables.

## Data Availability

The data that support the findings of this study are available upon reasonable request.
