# Supplementary material for: A dual functional theranostic microneedle patch for immunomodulation and real time monitoring in diabetic wound therapy
Source: Theranostics. 2026 May 1;16(12):6529–53. doi: 10.7150/thno.133451 (PMC13231991; doi:10.7150/thno.133451)
Supplement: Supplementary file 1 — Supplementary figures and tables. [file thnov16p6529s1.pdf]

Supporting Information

A dual functional theranostic microneedle patch for immunomodulation and real time monitoring in diabetic wound therapy

Shuai Fan[1,2,3,#], Junlong Zhong[1,2,#], Yeke Chen[3,5,#], Kang Chen[4], Zhiming Liu[1,2], Xinmin Yang[4], Wen Tan[4], Wenlong Tang[3,4], Wanhui Zhou[3,4], Degui Wu[3,4], Jiachao Xiong[1,2], Zhenhai Zhou[1,2*], Fanrong Ai[3,*], Kai Cao[2,3,4*]

[1]Orthopedic Hospital, The First Affiliated Hospital, Jiangxi Medical College, Nanchang University, Nanchang, Jiangxi 330209, China

[2]The Key Laboratory of Spine and Spinal Cord Disease of Jiangxi Province, Nanchang, Jiangxi 330006, China

[3]Bio 3D Printing Laboratory, School of Advanced Manufacturing, Nanchang University, Nanchang, Jiangxi 330031, China

[4]Department of Orthopedics, Affiliated Rehabilitation Hospital of Nanchang University, Jiangxi 330003, China

[5]The Second Clinical Medical School, Nanchang University, Nanchang, Jiangxi 33006, China

[#]Shuai Fan, Junlong Zhong and Yeke Chen contributed equally to this work.

*Corresponding author: Zhenhai Zhou (Email: zhouzhenhai2016@126.com); Fanrong Ai (Email: afr3755875@126.com); Kai Cao (Email: caokai@ncu.edu.cn, kaichaw@126.com)

Figures

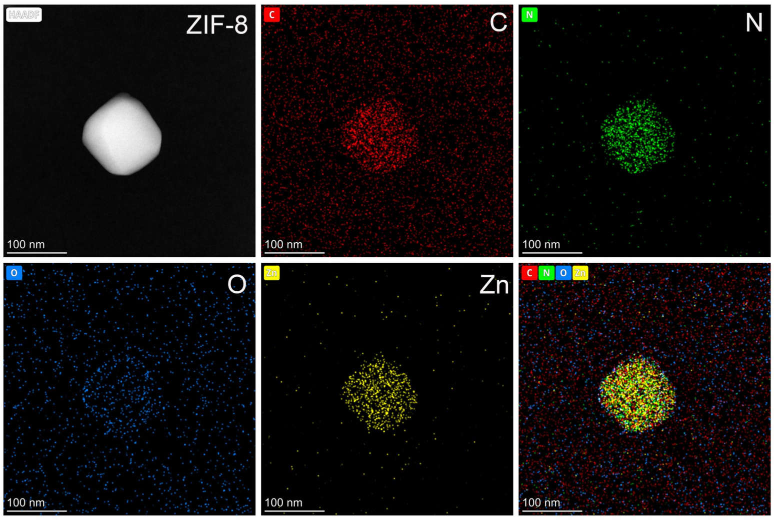

Figure S1. EDS spectrum of ZIF-8.

Element	C	N	O	Zn	Cu	Ga
Atomic fraction (%) ZIF-8	79.28	8.47	10.47	1.78	-	-
Atomic fraction (%) ZTCG	58.68	3.67	19.99	2.95	14.05	0.65

Figure S2. Elemental content comparison between ZIF-8 and ZTCG.

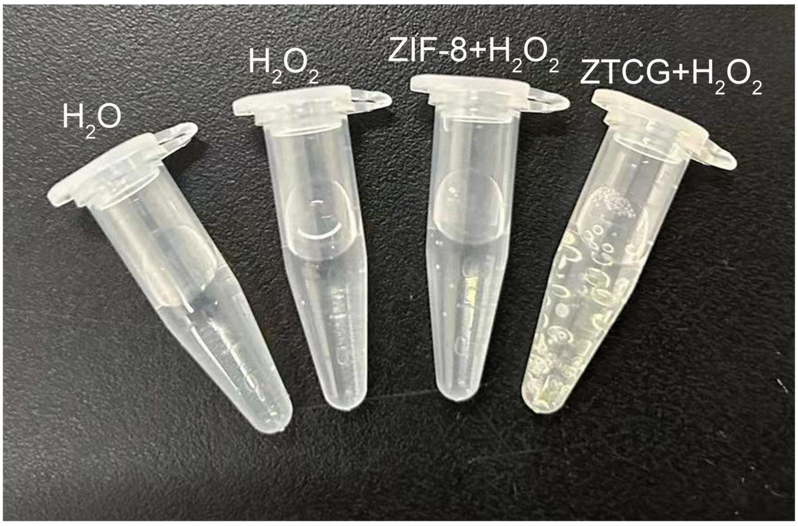

Figure S3. Bubbles observed after the incubation of H_2O_2 and ZTCG.

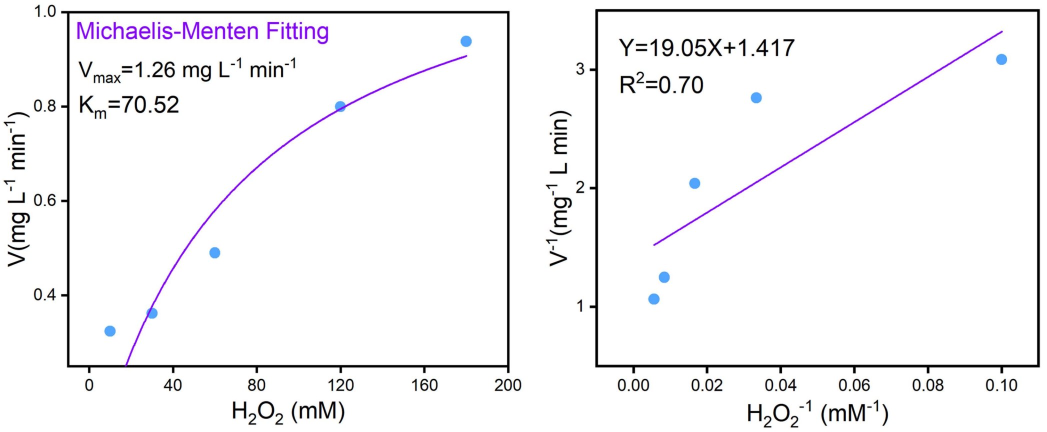

Figure S4. Michaelis-Menten kinetics and Lineweaver-Burk plotting for CAT-like activity of ZTCG.

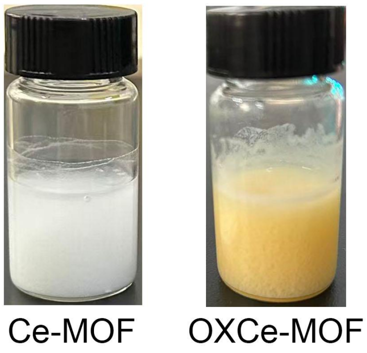

Figure S5. Colorimetric response images of Ce-MOF toward H_2O_2.

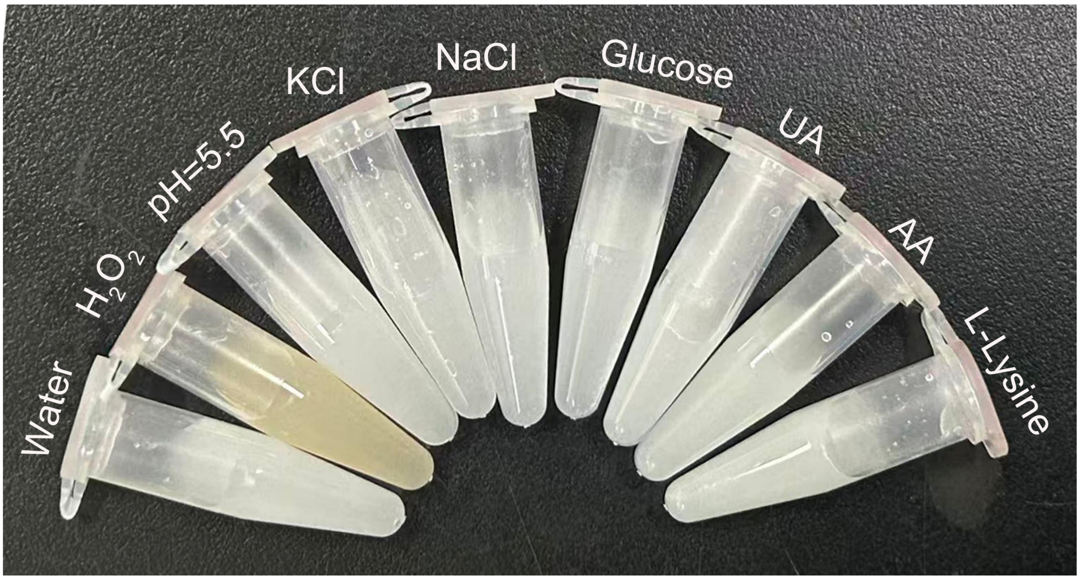

Figure S6. Color changes of Ce-MOF in solutions such as water, H2O2, acidic liquids (pH=5.5), potassium chloride (KCl), sodium chloride (NaCl), glucose, uric acid (UA),

ascorbic acid (AA), and L-Lysine.

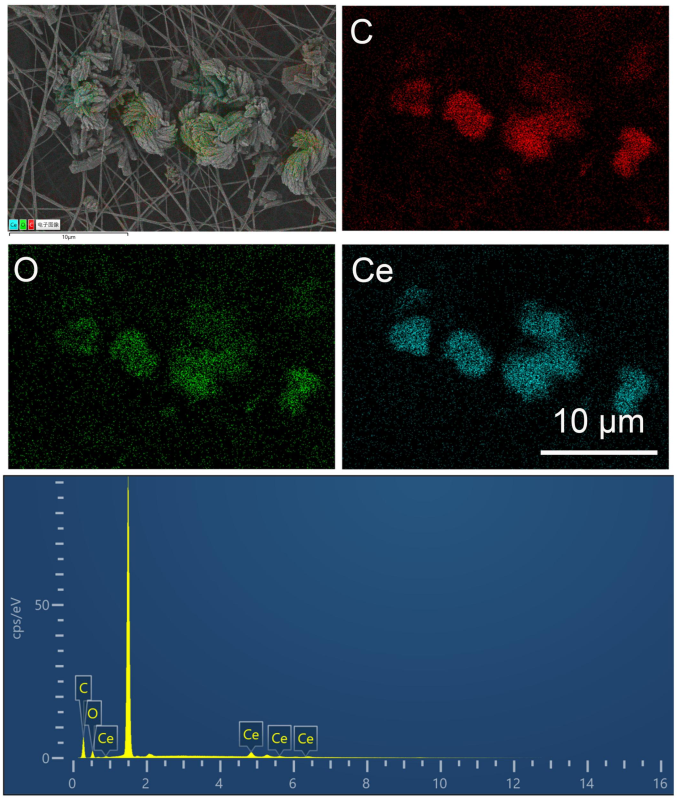

Figure S7. EDS spectrum of Ce-MOF/PCL.

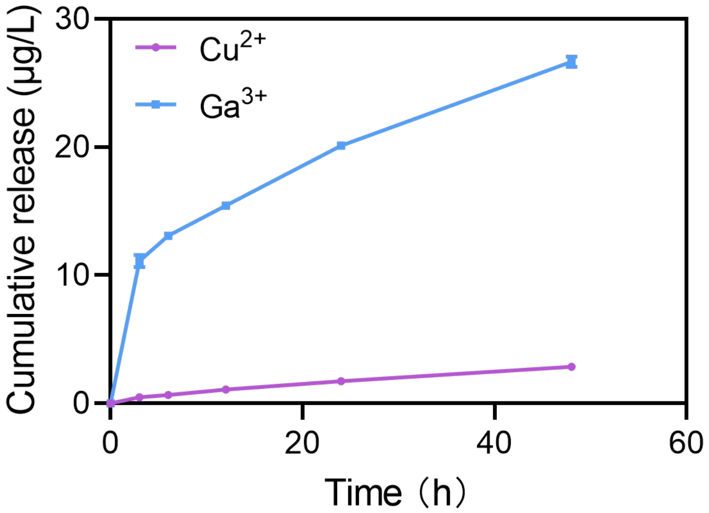

Figure S8. The cumulative Cu^{2+} and Ga^{3+} release from MNs@Z/CP.

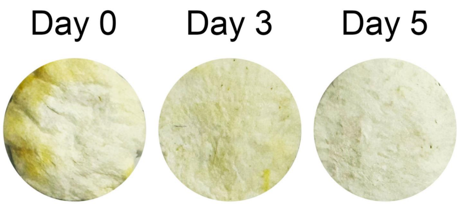

Figure S9. Color change images of MNs@Z/CP at different time points in the body.

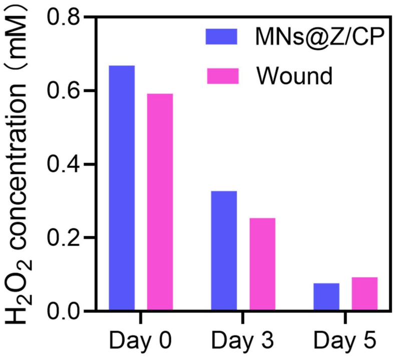

Figure S10. MNs@Z/CP and real-time quantitative determination of H_2O_2 at the wound site.

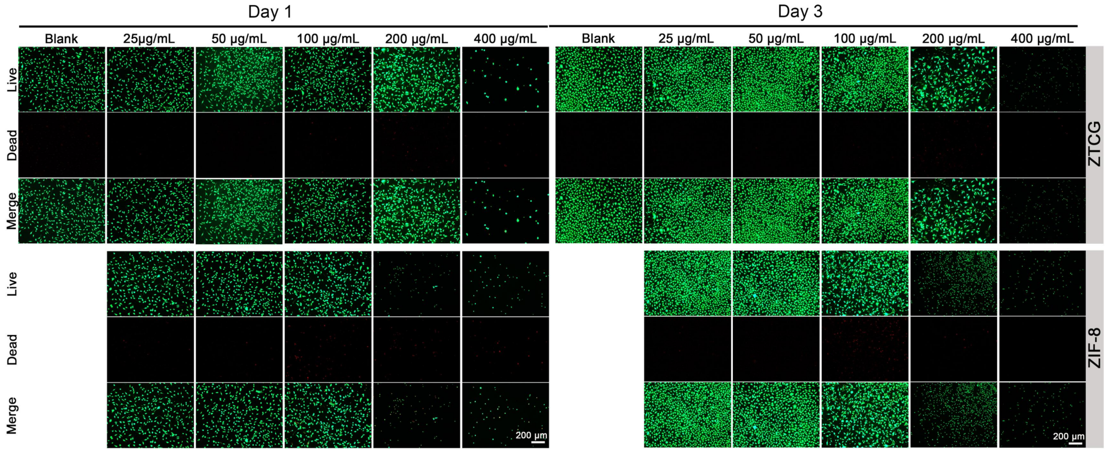

Figure S11. Live/dead staining assay of L929 cells cocultured with ZIF-8 and ZTCG for 1 and 3 days.

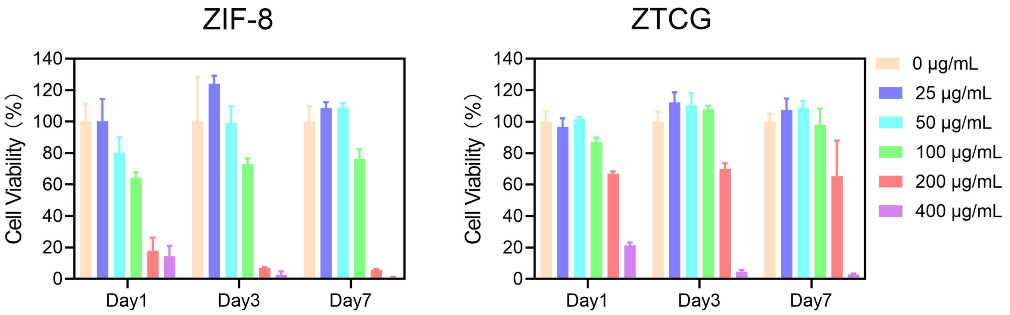

Figure S12. Cell viability of L929 cells cocultured with ZIF-8 and ZTCG for 1, 3 and 7 days. (n = 3).

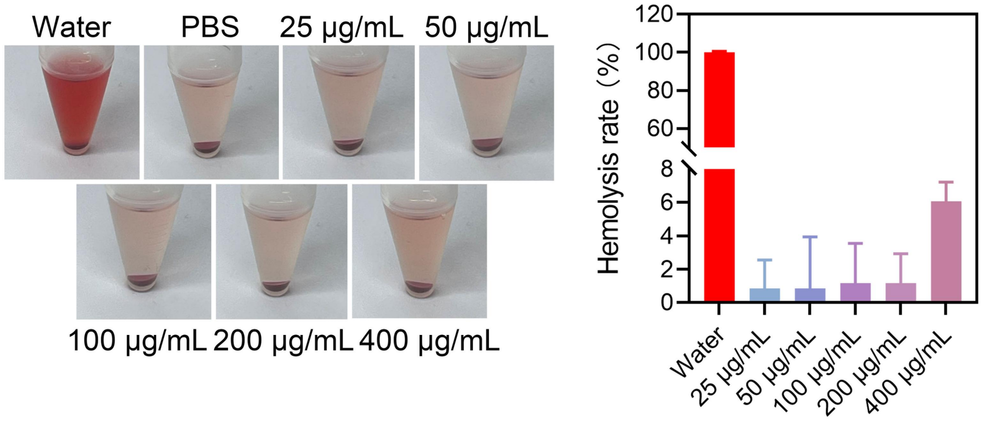

Figure S13. Hemolysis ratios of RBCs treated with different concentrations of ZTCG. (n = 3).

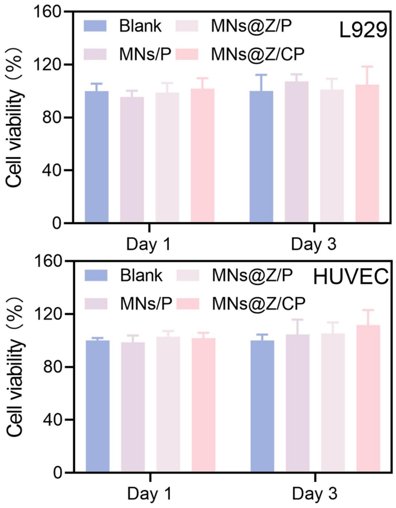

Figure S14. Cell viability of L929 cells and HUVECs cocultured with MNs@Z/CP for 1 and 3 days. (n ≥ 3).

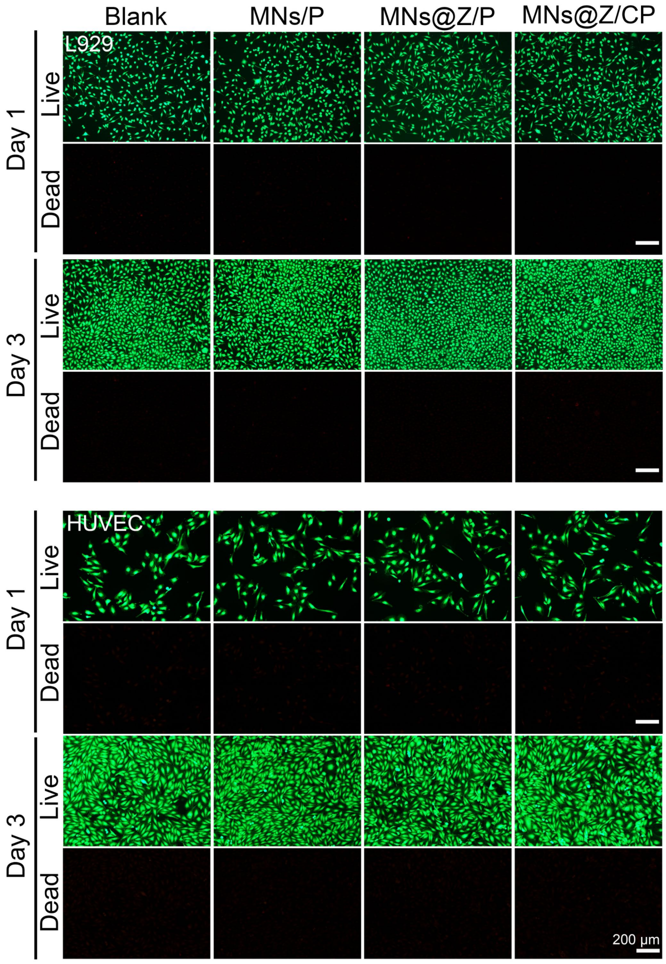

Figure S15. Live/dead staining assay of L929 cells and HUVECs cocultured with MNs@Z/CP for 1 and 3 days.

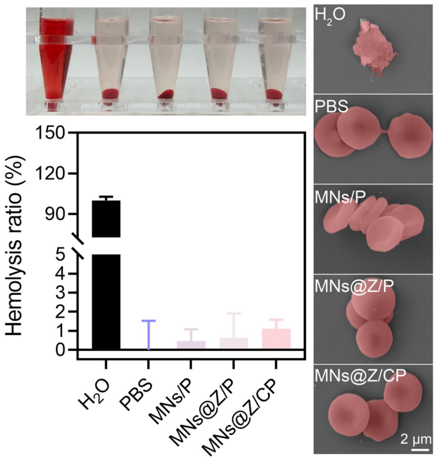

Figure S16. Hemolysis ratios of RBCs treated with MNs@Z/CP and SEM of RBCs treated with MNs@Z/CP. ($n \geq 3$).

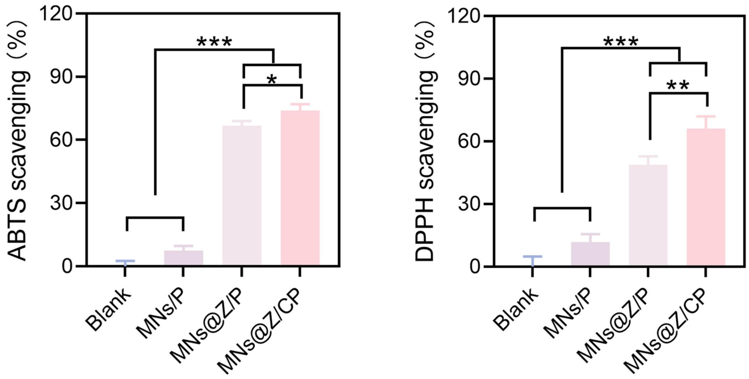

Figure S17. Scavenging efficiency of MNs@Z/CP against ABTS and DPPH. ($n = 3$).

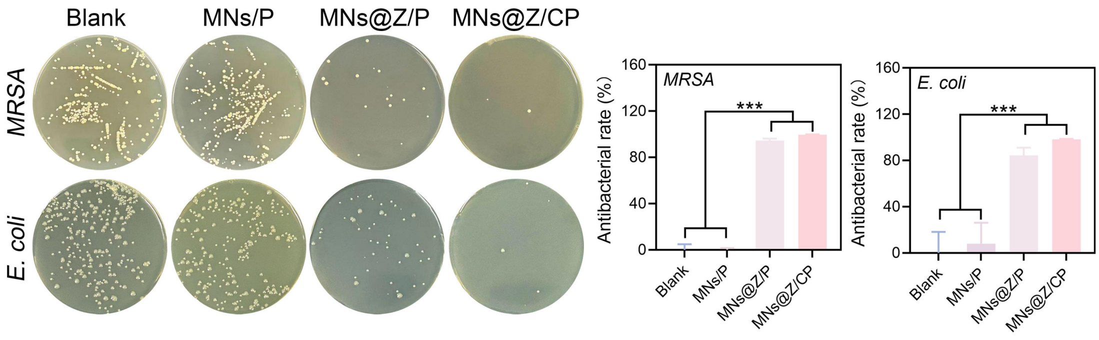

Figure S18. Representative plate colony counting images and quantitative analysis of *MRSA* and *E. coli* after treatment with MNs@Z/CP. ($n = 3$). ***$p < 0.001$

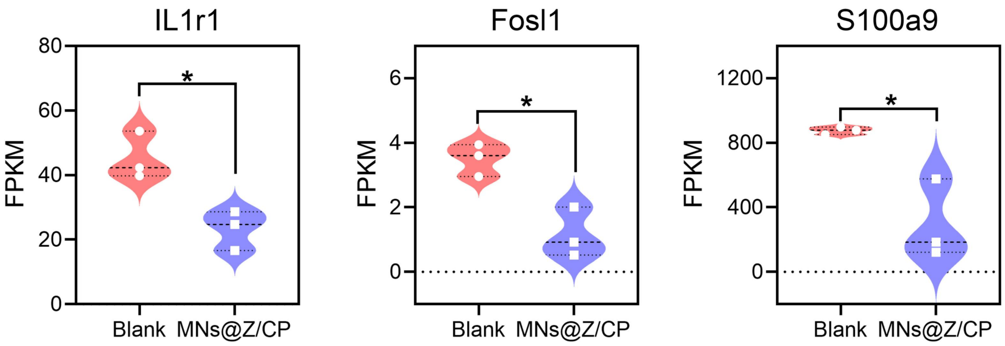

Figure S19. Fragments per kilobase million (FPKM) values of IL1R1, Fosl1 and S100a9. (n = 3). *p < 0.05

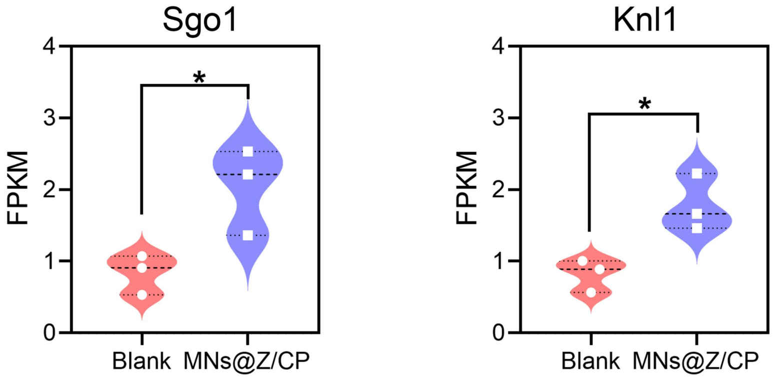

Figure S20. FPKM values of Sgo1 and Knl1. (n = 3). *p < 0.05

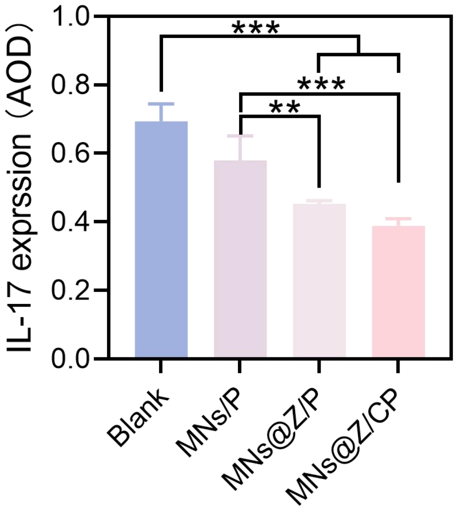

Figure S21. Quantitative analysis of the IL-17. (n =5). **p < 0.01, ***p < 0.001.

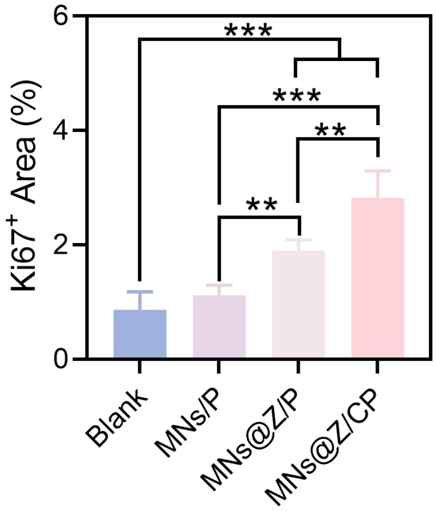

Figure S22. Quantitative analysis of the KI67^{+} area. (n =5). *p < 0.05, **p < 0.01, ***p < 0.001.

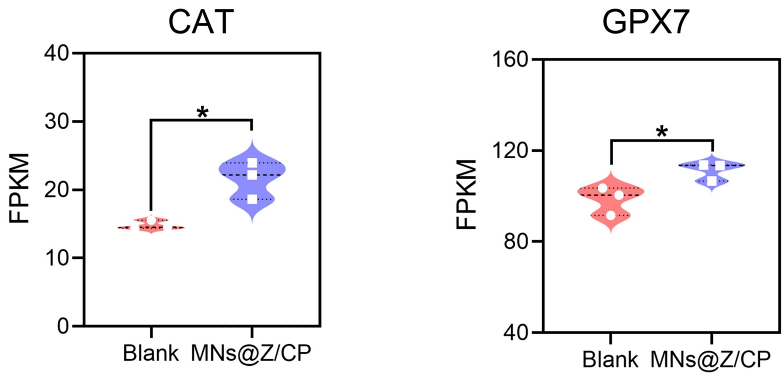

Figure S23. FPKM values of CAT and GPX7. (n = 3). *p < 0.05

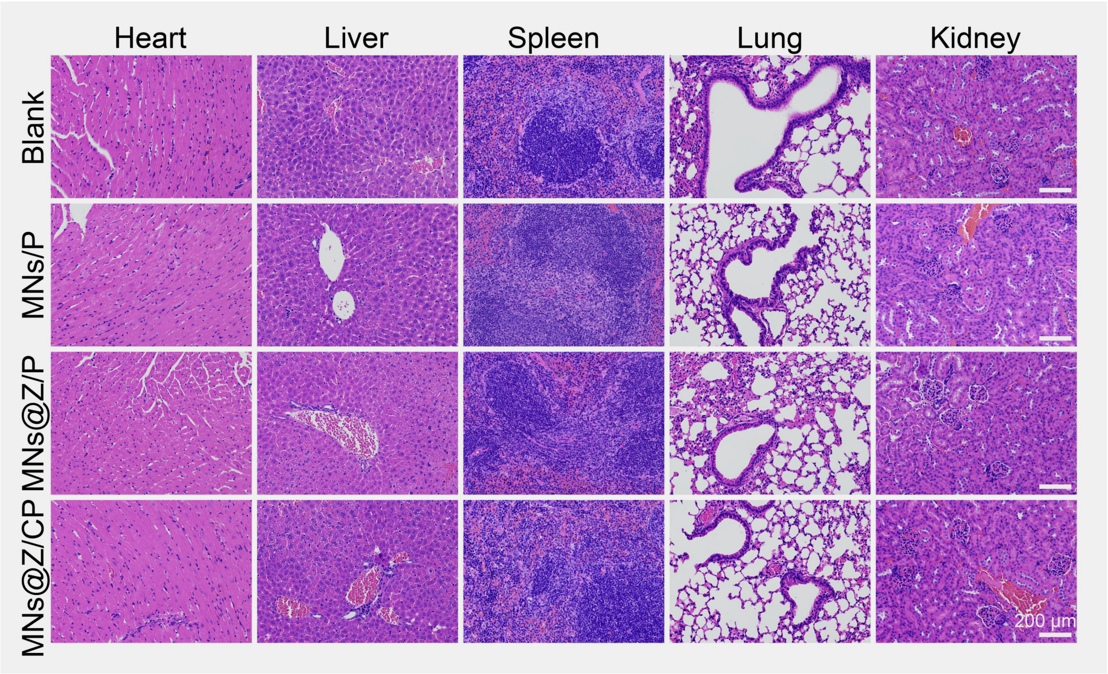

Figure S24. H&E staining images of vital organs in Blank, MNs/P, MNs@Z/P and MNs@Z/CP groups at 15 days.

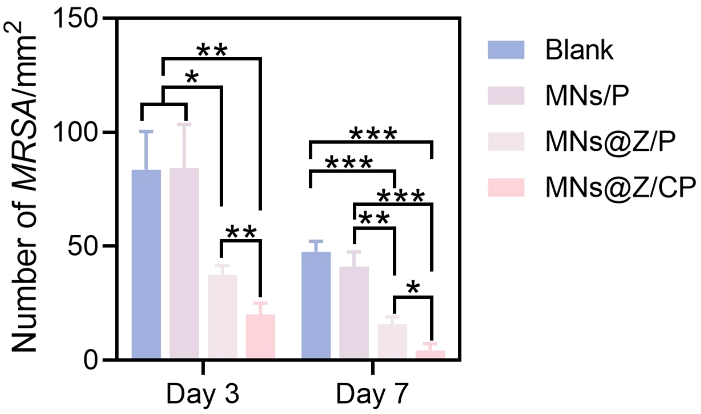

Figure S25. Quantitative analysis of the *MRSA* numbers according to the images of Giemsa staining. (n =5). *p < 0.05, **p < 0.01, ***p < 0.001.

Tables

Table S1. Primer sequences used for qRT-PCR.

Target gene	Forward sequence (5’-3’)	Reward sequence (5’-3’)
IL-6	TCCTACCCCAATTTCCAATGCT	AACGCACTAGGTTTGCCGAG
IL-10	CCTGGGTGAGAAGCTGAAGAC	CTTGTAGACACCTTGGTCTTGG
CD206	GGAGTGGCAGGTGGCTTATG	CACTGCTCGTAATCAGCCTCC
TNF-a	GCCGATGGGTTGTACCTTGT	TCTTGACGGCAGAGAGGAGG
INOS	AGCTCGGGTTGAAGTGGTATG	CACAGCCACATTGATCTCCG
Nrf2	ACACGAGATGAGCTTAGGGC	TCGGATCAATGCGAGCTGAG
Ho-1	CTGTCCAGTTGGTGTGGATAA	TCAGGCAGAGGGTGATAGAA
Keap1	TGAACGAGCTTCGCCTGAG	CGTGTAGGCGAACTCAATAAGC
CAT	CCAGCGACCAGATGAAGCAG	GTGACCTCAAAGTATCCAAAAGCA
Gapdh	CCTCGTCCCGTAGACAAAATG	TGAGGTCAATGAAGGGGTCGT

Table S2. The primary antibodies used in vitro experiments.

Target Protein	Supplier	Catalog No.	Application	Dilution
β-actin	Servicebio	ZB15001	Western blot	1:2000
INOS	Abcam	ab178945	Western blot	1:1000
			Immunofluorescence	1:500
CD206	Huabio	ET1702-04	Western blot	1:1000
	Abcam	ab300621	Immunofluorescence	1:50
Nrf2	Huabio	HA721432	Western blot	1:1000
			Immunofluorescence	1:100
HO-1	ABclonal	A19062	Western blot	1:2000
			Immunofluorescence	1:100
Keap1	ABclonal	A25951	Western blot	1:2000
NOX-1	ABclonal	A8527	Western blot	1:500
CAT	Huabio	ET1703-31	Western blot	1:1000
